# Supplementary material for: Genome‐wide prediction of synthetic rescue mediators of resistance to targeted and immunotherapy
Source: Mol Syst Biol. 2019 Mar 12;15(3):e8323. doi: 10.15252/msb.20188323 (PMC6413886; doi:10.15252/msb.20188323)
Supplement: Supplementary file 1 — Appendix [file MSB-15-e8323-s001.pdf]

Appendix

Genome-wide prediction of synthetic rescue mediators of resistance to targeted and immunotherapy

1 Appendix Figures Captions 2

2 INCISOR pipeline and SR network 8

2.1 INCISOR for SR- DU and DD, UD and UU types 8

2.2 Accommodating INCISOR for synthetic lethality 10

2.3 Verifying partner genes in predicted SR interaction does not lie in same chromosome 11

2.4 Check the robustness of parameter used in INCISOR 11

3 Analyzing and characterizing SR networks 11

3.1 Evaluating the predictive survival signal of the inferred SR networks 12

3.2 Evaluating the predictive survival signal of pan-cancer SR networks 12

3.3 Pancancer SL network and combined clinical impact of SL and SR 13

3.4 SR network with physical protein interactions 13

3.5 DU-SR network GO enrichment 14

3.6 DU and DD SR network sparsity and characteristic 14

3.7 SR interaction between pathways (pathway-SR network) 14

3.8 Functional similarity of DD-SR pairs 15

3.9 SR events increase in tumor with advanced cancer 15

3.10 TCGA (single nucleotide) mutation analysis 15

3.11 Breast cancer DU and DD SR interaction 16

4 Validation of SR prediction 17

4.1 Benchmarking INCISOR using published SR interactions 17

4.2 *In vitro* validation of INCISOR via shRNA inhibition of DD-rescuers 18

4.3 *In vitro* validation of INCISOR via drug response data DD-rescuer inhibitors 19

4.4 *In vivo* validation of DD-SR interactions using drug response data 20

4.5 Re-sensitizing patient-derived resistant cell lines to ALK/EGFR inhibitor 21

4.6 Experimental validation of DD-SR in head and neck cancer cell lines 21

4.7 Experimental validation of SR-based combinational therapies in head and neck cancer 23

4.7.1 Validating synergism of SR based drug combinations 23

4.7.2 Validating synergism of SR based drug combinations via siRNA experiments 23

5 Applications of DU-SR network 24

5.1 Drug-specific SR network 24

5.2 Tumor relapse prediction in ovarian and breast cancer 24

5.3 INCISOR successfully identifies drugs re-sensitizing resistant cell lines to the primary cancer drugs 24

5.4 Identification of in vivo synergistic drug combinations involving Benemitib (MEKi) 26

5.5 SR based supervised prediction of resistance to immune checkpoint blockades 26

5.5.1 Hugo et al. (Hugo et al, 2016) 26

5.5.2 Prat et al. (Prat et al, 2017) 27

5.5.3 Van Allen et al. (Van Allen et al, 2015) 27

5.6 Using SR strength to predict drug response in TCGA 27

6 SR based therapeutics opportunities 27

7 Limitation of INCISOR 28
8 Reference: 29

1 Appendix Figures Captions

Appendix Fig. S1. (a-e) Synthetic rescues functional truth tables: The truth tables of the four SR interaction types and the SL interaction. Each truth table denotes the cell viability states - *viable* (green), *non-rescued* (i.e., lethal -- red), and *rescued* (blue) - as a function of the activity state of each of the SR pair genes (down regulated, wild-type and up-regulated). The activity states are enumerated as 1 to state 9.: (a) **(**DU-SR): Down-regulation of a vulnerable gene is lethal but the cancer cell is rescued (retains viability) by the up-regulation of its rescuer partner; (b,d,e): Analogous functional truth tables for (DD, UD, and UU) SR types. (c) In a synthetic lethal interaction, in difference, the down-regulation of either gene alone is viable but the down-regulation of both genes together is lethal. **(f) The pan-cancer DU-SR network**, with red nodes denote vulnerable genes and green rescuer genes; the size of nodes is proportional to the number of interactions they have. **(g) The pan-cancer DD-SR networks**, with (red nodes denote vulnerable genes and green rescuer genes; the size of nodes is proportional to the number of interactions they have).

Appendix Fig. S2. (a-d) Network characterization of pancancer SR network: (a,b) Degree distribution of DU-SR network for rescuer genes **(a)** and vulnerable genes **(b)**. **(c,d)** : Degree distribution of DD-SR network for rescuer genes **(c)** and vulnerable genes **(d). (e) Functional similarities between gene pairs in DD SR network.** Comparison of functional similarities between (i) SR: DD-SR network (ii) Random-pair: network generated by random pairing between protein coding genes degree distribution similar to DD-SR network (iii) shuffled-pair: network generated by shuffling pairing of DD-SR network. Functional similarities of a pair were evaluated in terms of (i) GO similarity (left panel), (ii) distance between the partner gene in PPI network (middle panel), and (iii) distance between the partner gene in STRING network (right panel). The significance of difference of each of above measure between SR and random and shuffled controls are displayed. **(f) Pairwise gene enrichment analysis**: The figure shows relationship between the biological processes to which many vulnerable genes belong (red) and the biological processes to which many of their rescuer partners belong. Edges between a vulnerable process and a rescuer process represent the enrichment of SR interactions between the vulnerable and rescuer processes higher than expected. **(g,h) The functional activity of DU-SR increases with advanced cancer**. **(g)** The trend of number of functionally active SRs (green) and random gene pairs (red) as cancer progresses. **(h)** The number of rescued inactive vulnerable genes with varying number of active rescuers (from single rescuer with darkest blue line

to five rescuers with the lightest blue line) as cancer progresses. **(i) DU-SR network predicts patient's survival in an independent dataset.** A Kaplan-Meier (KM) analysis comparing the survival of patients whose tumors have many functionally active SRs (top 10 percentile (N=200), *rescued*) to those with a few (bottom ten percentile (N=200), *non-rescued*). The difference in the areas under the curve between rescued (blue) and non-rescued (red) samples (ΔAUC) and their log rank p-values are denoted, in addition to Hazard ratios and their significance obtained from a Cox regression. **(j)** Survival prediction by integrating both SL and SR networks. The subset of non-rescued patients in Figure S2i that also have many co-inactive SLs (top 10 percentile (N=87); Appendix 3.3) shows remarkably better survival than the subset of rescued patients that additionally have very few co-inactive SLs (bottom ten percentile (N=158)). **(k) DD-SR networks predict cancer patient's survival:** A Kaplan-Meier (KM) analysis comparing the survival of patients whose tumors have many functionally active DD SRs (top 10 percentile (N=200), *rescued*) to those with a few (bottom ten percentile (N=200), *non-rescued*). The difference in the areas under the curve between rescued (blue) and non-rescued (red) samples (ΔAUC) and their log rank p-values are denoted. **(l) Pan-cancer clinical significance of SR network**. X axis shows 23 different cancer types (with >50 samples in each type), and Y axis shows the fraction of significant pan-cancer SR interactions in each cancer type. Pan-cancer TCGA dataset was divided into two halves. DU-SR network was identified by applying INCISOR using one half of the data, and clinical significance was determined in the other half of the data. **(m,n) PPI-SR (DU) network: (m)** The pan-cancer DU-SR network mediated by protein-protein physical interactions (with red nodes denote vulnerable genes and green rescuer genes; the size of nodes is proportional to the number of interactions they have). **(n) The PPI-SR network successfully predicts cancer patient's survival.**

Appendix Fig. S3. Validation of INCISOR predicted SR interactions using seven published datasets **identifying DU-SR rescuers** of seven drugs(IFong et al, 2015; Mills et al, 2013; Rathert et al, 2015; Stuhlmiller et al, 2015; Zhang et al, 2016). INCISOR's prediction accuracy in identifying the rescuers of each of following seven drugs are quantified using receiver operator curves (ROC) and precision-recall (P-R) curves: ABT-737(Mills et al, 2013) (a,e), BET-inhibitor(IFong et al, 2015; Rathert et al, 2015)(b,f), Lapatinib(Stuhlmiller et al, 2015)(c,g), Estrogen receptor inhibitor(Zhang et al, 2016)(d,h), Vemurafenib (Hugo et al, 2015)(i,j), BRAF inhibitor(k,l) and Tamoxifen(Gonzalez-Malerva et al, 2011)(m,n). **(o) Summary figure of validation of INCISOR predicted SR interactions using** four published datasets **identifying DU-SR rescuers** of four drugs (IFong et al, 2015; Mills et al, 2013; Rathert et al, 2015; Stuhlmiller et al, 2015; Zhang et al, 2016). AUCs quantifying INCISOR accuracy in identifying the rescuers of each of four drugs screened, including ABT-737, BET inhibitor, Lapatinib and an Estrogen receptor inhibitor. (p) **The contribution of each step of INCISOR to its overall prediction, assessed via using**

four published datasets identifying DU-SR rescuers of four drugs(IFong et al, 2015; Mills et al, 2013; Rathert et al, 2015; Stuhlmiller et al, 2015; Zhang et al, 2016)**.** The coefficients estimated (Y-axis) by a multi-variate logistic regression for each INCISOR step (X-axis), for the same four datasets. The significance of the coefficients is displayed ("*" < 0.05, "**" < 0.01, "***" < .001).

Appendix Fig. S4. Validation of INCISOR predicted SR interactions using published in vivo and in vitro data. (a,b) INCISOR's performance in identifying drugs that mitigate resistance to EGFR or ALK inhibitors (Crystal et al, 2014). **(a)** The association between the INCISOR scores (Y-axis, Methods) and the experimentally observed drug efficacy (X-axis). **(b)** An ROC curve depicting the accuracy of INCISOR to identify drugs that sensitize the cells derived from resistant to EGFR or ALK inhibitors. **(c) Targeting predicted DD rescuers enhances tumor size in mouse xenografts** (Gao et al, 2015): The X axis shows the tumor size increase/decrease following DD-rescuer inhibition by drug treatment in all *mice xenografts* (*general effect)* where there is no copy number loss of their corresponding vulnerable gene. The Y axis shows the corresponding *conditional effect* of DD-rescuer pharmacological inhibition, measured only in the xenografts where the *corresponding vulnerable gene is lost*. As predicted, targeting predicted rescuers (red) results in significantly larger tumors in the conditional cases (where the vulnerable gene is lost, and the rescue indeed happens, Y axis) than in the general cases (where the vulnerable gene is not loss, and no rescue occurs, X-axis) ((Wilcoxon P-value <2.2 E-16)). This differential effect is generally not observed when targeting random control genes. Circles denote pairs that have a significant (P-value < 0.01) and crosses denote insignificant rescue effects. The Inset displays the synthetic rescue effects of HSP90 treatment. **(d)** Same as **(c)**, except the PDX tumor growth displayed in terms of cumulative tumor growth. **(e) Progression free survival (PFS) decrease in PDX with synthetic rescue**: The figure compares the PFS of the general (vulnerable gene not lost) and conditional (vulnerable gene lost) PDXs following the drug treatment (DD-rescuer inhibitor). **(f,g) Validation of SR DD network using large-scale drug treatments in cell lines CTRP(Basu et al, 2013) and CCLE (Cheung et al, 2011)**: Density distribution of the *conditional* normalized IC50 of 145 drugs in CTRP **(f)** and 24 drugs in CCLE **(g)** rescuer inhibitors in cell lines having a copy number loss of corresponding vulnerable genes for predicted DD-pairs (yellow line) and for random pairs (grey line). The IC50 values of the *conditional* IC50 DD-pairs are significantly higher than that of the random pairs, testifying to the predicted rescue effects. The figure additionally displays the normalized *conditional* IC50 of DD-rescuer inhibitors for the top 500 DD-interactions, with red and green dots respectively denoting significant and insignificant decrease of anti-proliferative effects of the drugs, testifying to predicted rescue effects. The significance of the rescue effect is quantified by the deviation of conditional IC50s from the expected value (shown in vertical the black line). **(h) Validation of predicted DD-SR interactions employing in vitro shRNA knockdowns (KD)(Marcotte et al, 2016)**: The X axis

shows the general phenotypic effect of DD-rescuer inhibition across all cell lines where there is no copy number loss of their corresponding vulnerable gene. The Y axis shows the corresponding conditional effect of DD-rescuer down regulation, measured only in the cell lines where the corresponding vulnerable gene is lost. The phenotypes measured are the post knockdown (KD) essentiality of shRNA (Marcotte et al, 2016).

Appendix Fig. S5. (a-d) Experimental shRNA screening validates (DD) rescue effects of mTOR. **(a)** Summary of pooled shRNA experiment. Time points, treated and control samples are explained in the figure. **(b-d) Rescue effect of top 11 predicted DD-SRs of mTOR.** The Y axis shows the cell count fold change in Rapamycin-treated vs. untreated cells (i.e., in the rescued versus the non-rescued state) for the top 11 predictions. Significance was quantified using a one-sided Wilcoxon rank-sum test over three technical replicates with at least two independent shRNAs knockdowns per each gene. For 8 of these KDs, at least two shRNA individually show the rescue effect. The black horizontal line indicates the median effect of Rapamycin treatment as a reference point. **(c)** Cell viability following shRNA(red) and shRNA+Rapamycin (blue) treatments are displayed. 11 predicted DD vulnerable partners of mTOR are knocked down using shRNA. Several sets of control genes (5 genes in each set that is the total of 25 genes) that are not the predicted SR partners of mTOR were additionally knocked down and screened in a similar manner for comparison. These control sets include proteins known to interact physically with mTOR, computationally predicted SL and synthetic dosage lethal (SDL) partners of mTOR, predicted DD-SR vulnerable partners of non-mTOR genes, and DD-SR predicted rescuer partners of non-mTOR genes. **(d)** Same as Appendix Fig. S5c, except the cellular viability is shown per shRNA. **(e,f) Procedure followed for experimental validation of predicted synergistic SR-based combinational therapies in head and neck cancer via a representative combination(Dasatinib + BYL19) in a representative cell line (HN12)**: **(e)** Dose matrix combinatorial drug treatment (48h) with a KIT inhibitor (Dasatinib) and an inhibitor of a predicted rescuer PIK3CA (BYL719) in HN12 cells. Numbers indicate % Cell Viability (n = 3) **(f)** Fa-CI curves created based on the matrix data. Dasatinib: BYL719 dose ratios are indicated by green and red curves. The Y axis displays the combination index (CI; synergism CI < 1, additivity effect CI = 1, antagonism CI > 1) at different levels of growth inhibition (Fraction affected, X axis). Similar procedure was applied to estimate synergism for other combinations in all cell lines tested (Appendix Fig. S6,7).

Appendix Fig. S6 (a-g). Experimental validation of proposed SR-based combinational therapies in head and neck cancer: Dose matrix combinatorial drug treatment (48h) with seven predicted drug combinations (displayed a top of each panel) in five head and neck cancer cell lines (Cal27, Cal33, HN12, Detroit 562 and SCC47). Numbers indicate % Cell Viability (n = 3). Drug combination tested is displayed at the top of each panel. Cell line used are displayed in each subpanel.

Appendix Fig. S7(a-g). Experimental validation of proposed SR-based combinational therapies in head and neck cancer (contd.): Fa-CI curves created based on the matrix data of Appendix Fig. S6. Drug combination dose ratios are indicated by green and red curves. The Y axis displays the combination index (CI; synergism CI < 1, additivity effect CI = 1, antagonism CI > 1) at different levels of growth inhibition (Fraction affected, X axis). Each panel displays corresponding drug combination and cell line. The experiments that failed to show synergism as per on Fa-CI analysis are marked red. h. **Experimental validation of seven predicted synergistic SR-based combinational therapies in head and neck cancer.** Ref. to main text Fig 2b.

Appendix Fig. S8. Experimental validation of proposed SR-based combinational therapies in head and neck cancer via siRNA and drug treatment: (a-h) Sensitization of four cell-lines (Cal27, HN12, Detroit 562 and SCC47) to two combinations (Dasatinib treatment & PIK3CA-siRNA; BYL719 & mTOR−siRNA). Drug response curves (DRC) (Y-axis) for Dasatinib/BYK719 cells, following the knockdown of PIK3CA/mTOR with siRNA versus a control siRNA KDs (cell lines and combinations are displayed above each panel). **(i)** The increase in sensitivity of the four cell-lines to BYL719 treatment with siRNA knockdown of mTOR and increase in its sensitivity to Dasatinib treatment with siRNA knockdown of PIK3CA: Percentage increase in sensitivity of the primary therapy (Y-axis) following the combinational treatment denotes the decrease in IC50 of the primary therapy observed in the combination setting relative to the primary therapy alone.

Appendix Fig. S9. Harnessing SR interactions to Identify Drugs Sensitizing Cancer Cells to the Primary Treatments: (a,b) INCISOR's performance in identifying drug combinations re-sensitizing resistant cell-lines in melanoma dataset for 108 drugs(Friedman et al, 2015), in terms of **(a) ROC and (b) precision recall curves. (c-f) *In vivo* survival improvement with combinational treatments involving the MEK inhibitor Binimetinib and SR-predicted drugs in mouse PDXs.** The Kaplan-Meier (KM) analyses compare the survival of mice xenografts treated with the predicted combinations (blue) vs those treated with Binimetinib alone (red) and with drugs inhibiting the predicted DU(c-e) or DD (f) rescuer genes alone (green). The difference in the areas under the curve (ΔAUC) and log rank p-values are denoted.

Appendix Fig. S10. (a-d) Rescuer activation associated with the vulnerable gene inactivation due to somatic mutations. (a) SCNA levels of rescuer genes in the samples where their vulnerable partner is mutated (red) vs not-mutated (blue). (b) Rescuer activation per each vulnerable gene mutation. The horizontal axis lists vulnerable genes with somatic mutations in TCGA samples, and the vertical axis denotes

the significance of increase in the rescuer gene activity (mRNA:red, SCNA:blue) between samples with and without vulnerable gene mutations. (c) Rescuer activation per each rescuer (in difference from (b)), all vulnerable partners of a rescuer were considered). The horizontal axis lists rescuer genes with somatic mutations in TCGA samples and the vertical axis denotes the significance of rescuer gene-activity (mRNA:red, SCNA:blue) between samples with vs. without vulnerable gene mutations. (d) The KM plot depicts the aggregate clinical predictive power of rescuers of CDH11 gene, among patient with CDH11 mutation). **(e)** The **drug-DU-SR** network includes 224 interactions between 48 drug cancer drug target genes (red) and 105 rescuer genes interacting with them (green). The drugs (purple) are linked to their targets. **(f) The DU-SR network identifies key molecular alterations associated with tumor relapse after Taxane treatment.** Post-treatment expression of the predicted rescuer genes in the relapsed tumors (red) compared to their activation level in pre-treatment primary tumors (green). Significantly altered genes (10 out of 14, all in the predicted direction) are marked by stars (one-sided Wilcoxon rank-sum $P<0.05$). **(g) Pre-treatment rescuers expression successfully predicts future relapse among initial responders in breast cancer.** An ROC plot in breast cancer shows the prediction accuracy obtained by a linear SVM (AUC=0.74) compared to the accuracy obtained based on 13 random genes (red line, AUC=0.57). **(h,i) The transcriptomic alterations of rescuer genes on-treatment PDL1 & CTLA4 blockade in on-treatment tumor biopsies:** on (vs pre) treatment expression changes of DU and DD rescuers during anti-PD1 **(h)** or anti-CTLA4 **(i)** therapy regiment. Each panel displays the expression fold change of each predicted rescuer gene (rows) for different tumor samples (columns) and the P-value of over-all paired Wilcoxon test of the expression changes observed in paired samples. **(j) A comparative analysis of the performance of supervised (red) and unsupervised (blue) SR-based predictors in predicting patients drug response (TCGA).** The area under curve (Y-axis) displays the predictive performance of two methods. The SR-based supervised predictor was constructed by using expression and SCNA of DU rescuer as features to train Random forest. The AUCs of supervised predictors were determined using two-fold cross validation.

Appendix Fig S11. (a) Robustness of thresholds used in KM analyses. KM analysis comparing the survival of patients whose tumors have many functionally active SRs (*rescued*) to those with a few (*non-rescued*). Different thresholds (5%, 15% and 25%) were tested to check the robustness of thresholds to identify rescued and non-rescued tumors. The differences in the areas under the curve between rescued and non-rescued samples (ΔAUC) and their log rank p-values are denoted for each threshold, in addition to hazard ratios and their significance obtained from a Cox regression. **(b-d)** Survival prediction: Breast cancer DU-SR (a) and DD-SR (b) network predict patient's survival in an independent Metaberic dataset. A KM analysis comparing the survival of patients whose tumors have many functionally active SRs (top

10 percentile (N=200), rescued) to those with a few (bottom ten percentile (N=200), non-rescued). The difference in the areas under the curve between rescued (blue) and non-rescued (red) samples (ΔAUC) and their log rank p-values are denoted, in addition to hazard ratios and their significance obtained from a Cox regression. (d) The Y axis displays logrank p-values per drug, denoting how well the response is predicted by DU-SR network in terms of survival difference between predicted responder and non-responders. **(e) Survival prediction of SL (ISLE (Lee et al, 2018))**. The KM plot shows that patients with high SL-scores (solid line) have better prognosis than those with low SL-scores (dashed line), using five different SL score thresholds, namely top/bottom 10% (red), 20% (orange), 30% (green), 40% (blue), and 50% (purple). **(f)** Overlap of predicted (DU) SR pairs using INCISOR with gene activation thresholds of 0.33 and 0.25 **(g) Using SR strength to predict cancer drug response in patients.** The Y-axis denotes the sum of SR strengths (SR-scores) of the DU-SR upregulated rescuers in tumors of responders (orange) and non-responders (green). Significant results are marked by stars. **(h) Western blot analysis of signaling events in HN12 after knock down of the *PIK3CA* and mTOR.** Top, HN12 (left) and SCC47 (right) cells were transfected with negative control or the corresponding *PIK3CA* siRNAs for 72 hours, and lysates were analyzed as indicated. Bottom, HN12 (left) and SCC47 (right) cells were transfected with negative control or the corresponding mTOR (*FRAP1*) siRNAs for 72 hours, and lysates were analyzed as indicated. In every case, the '-' indicates control cells without transfected siRNA.

2 INCISOR pipeline and SR network

2.1 INCISOR for SR- DU and DD, UD and UU types

During cancer progression, fitness-reducing alterations in a particular gene may be compensated by subsequent alterations in the activity of another gene, restoring cancer progression and proliferation. In this type of genetic interaction, we term the former gene a *vulnerable* gene, the latter gene a *rescuer* gene, and the functional relation between them a *synthetic rescue* (SR). There are potentially four types of SRs: (1) down-regulation of both the vulnerable and the rescuer gene (DD); (2) down-regulation of the vulnerable gene and up-regulation (i.e., over-activation) of the rescuer (DU); (3) up-regulation of the vulnerable gene and down-regulation of the rescuer (UD); and (4) up-regulation of both vulnerable and rescuer genes (UU) (see Appendix Fig. S1a-d).

For a pair of gene (vulnerable-rescuer) with DU-SR interaction between them, we define its *non-rescued* state as molecular state when a cancer cell is at stress due to down-regulation of the vulnerable

gene, but the cell is not rescued because the rescuer gene is not up-regulated (Appendix Fig. S1a, Dataset Table E1). Similarly, its *rescued* state is defined as the case where the vulnerable is down regulated but the cellular stress is relieved by rescuer up-regulation. As shown in **Dataset Table E1**, different molecular states represent *rescued* and *non-rescue*d state for different type of SR interactions (Appendix Fig. S1a-d).

type	***rescued* state**			***non-rescued* state**		
	Activity States	*Vulnerable gene*	*Rescuer gene*	*Activity States*	*Vulnerable gene*	*Rescuer gene*
DU	3	Down-regulated	Up-regulated	1,2	Down-regulated	Not Up-regulated
DD	1	Down-regulated	Down-regulated	2,3	Down-regulated	Not Down-regulated
UD	7	Up-regulated	Down-regulated	8,9	Up-regulated	Not Down-regulated
UU	9	Up-regulated	Up-regulated	7,8	Up-regulated	Not Up-regulated

Dataset Table E1: Definition of rescued and non-rescued states in each of the four types of SR interactions. The pair-wise gene activity states (based on Appendix Fig. S1a) of each rescued/non-rescued state are also displayed.

We applied INCISOR to mine TCGA tumor molecular and survival data of 8,749 cancer patients across 28 different cancer types, and analyze genome-wide shRNA screens composed of 2.2 million measurements in 220 cancer cell lines (Cheung et al, 2011; Cowley et al, 2014; Marcotte et al, 2012; Marcotte et al, 2016).

Figure 1c and Appendix Fig. S1f displays predicted DU-SR network, composed of 1033 interactions between 1109 genes, with 614 vulnerable and 598 rescuer genes. Dataset Table E2,3 shows for each DU-SR interaction in the network, pan-cancer clinical significance and the significance in each of 28 cancer types.

Appendix Fig. S1g displays DD-SR network, composed of 1967 interactions between 1084 genes, with 645 vulnerable and 597 rescuer genes. Dataset Table E4,5 shows for each DD-SR interaction in the network, pan-cancer clinical significance and the significance in each of 28 cancer types.

2.2 Accommodating INCISOR for synthetic lethality

We modified INCISOR to predict synthetic lethal (SL) interactions (Appendix Fig. S1c). Specifically, we adopted different statistical screens in INCISOR to identify SL that occurs in a patient's tumor and is likely to have a therapeutic value. The resultant approach, termed **ISLE** (Identification of clinically relevant Synthetic Lethality), which is currently under review, adopts following modifications over INCISOR:

(1) *In vitro* screening: By definition, it is expected that gene A will be essential only when its SL partner gene B is inactive in a given cancer cell line. Accordingly, ISLE uses genome-wide shRNA/CRISPR screening to identify a gene pair A and B as candidate SL partners if participating genes show conditional essentiality based on its partner's low gene expression/SCNA. In addition, ISLE takes the experimentally identified published SL interactions from double knockout or isogenic cell line experiments to create the initial pool of candidate SL interactions.

(2) *Molecular survival of the fittest* (SoF): A SoF-SL-pattern between two genes (A and B) denotes that samples, where both gene A and B are inactive, are significantly less frequent than expected. Analogous to SR identification, we employ a simple hypergeometric test to identify depletion of samples in the bin, where both genes A and B are down-regulated, followed by standard false discovery correction.

(3) *Patient Survival screening*: Co-inactivated of a SL gene pair (A and B) in a tumor is lethal, and hence patients with co-inactive SL gene pair will have better survival. Accordingly, ISLE employs a Cox multivariate regression analysis to identify candidate SL partners whose co-inactivation is associated with improved survival to a greater extent compared to the additive effect of the individual gene inactivation of the candidate SL partners. We also control for various confounding factors including cancer types, sex, race, genomic instability, and age.

(4) Phylogenetic screening: Analogous to SR phylogenetic screen

ISLE identified 2,326 SL interactions between 2,153 genes. And none of these interactions overlaps with predicted DU-SR interactions by INCISOR.

2.3 Verifying partner genes in predicted SR interaction does not lie in same chromosome

INCISOR uses gene expression and SCNA data from patient tumors to infer SR interactions. This may lead to the inference of false positive SRs due to correlations between the expression of different genes and from correlations in the copy number alterations of proximally located genes. To demonstrate that SR identified by INCISOR are not confounded by genomic linkage arising due to such false positive inference, we verified that the SR interactions are not biased towards genes lying on the same chromosome as follows.

We first estimate percentage of SR interactions where both the partnered genes will lie in the same chromosome by random chance. This was estimated by creating gene pairs selected randomly from all protein coding genes. For 5.4% among all randomly selected pairs, partnered genes lie in same chromosome. Out of 1195 predicted DU-SR interactions, 2.9% lie in same chromosome. Out of 1195 predicted DU-SR interactions, 5.9% lie in same chromosome, which is not significantly greater than 5.4% expected by the random chance (Hypergeometric P > 0.1).

2.4 Check the robustness of parameter used in INCISOR

INCISOR uses the threshold of 1/3 (2/3) across the samples in each cancer type for down-regulation (up-regulation) of genes. It seems a natural choice since we consider three levels of activity (down, WT and up) for each gene, as usually is conceived by biologists. We tested the robustness of these cut-offs (thresholds) as follows:

We rerun INCISOR by using a 25% threshold for gene activation (i.e, top 25% of samples were considered over-expressed, while the bottom 25% were considered down-regulated and the rest 50% as WT). The result is shown in Appendix Fig. S11f. The overlap for two thresholds was significant (Hypergeometric P < 2.2E-16).

3 Analyzing and characterizing SR networks

In this section, we will provide the first a brief overview of utility of SR networks in predicting clinical prognosis (survival) and other characterization of the networks. Then we discuss in details each of the characteristics.

We turned to application of SR networks to predict survival. We applied INCISOR to mine TCGA tumor molecular and survival data of 8,749 cancer patients across 28 different cancer types, and analyze genome-wide shRNA screens composed of 2.2 million measurements in 220 cancer cell lines (Cheung et al, 2011; Cowley et al, 2014; Marcotte et al, 2012; Marcotte et al, 2016). We studied the ability of the SRs identified to predict patient survival using the transcriptomics and copy number data of individual tumors in

an independent METABRIC Breast Cancer (BC) dataset(Curtis et al, 2012) in Appendix Section 3.1, 3.2. Combining SR interactions with SL interactions further improves the patient survival predictive power (Appendix Section 3.3). The analysis of subset of DU-SR interactions having direct protein-protein interactions are detailed in Appendix Section 3.4.

Gene enrichment of DU-SR network is provided in Section 3.5. Analyzing the DU-SR network revealed: (i) it is a scale-free network (Appendix Fig. S2a-d, Details in Section 3.6), (ii) partnering genes are closer in Gene Ontology tree network (Appendix Fig. S2e, Details in Appendix Section 3.7), (iii) a certain pathway genes have higher propensity to rescue by the genes that belong to specific pathways (Appendix Fig. S2f, Details in Appendix Section 3.8), and (iv) the number of functionally active SR interaction in tumor increases with advanced cancer stages (Appendix Fig. S2g,h, Details in Appendix Section 3.9), (v) the predicted DU-rescuer genes are specifically up-regulated in patients' tumor with mutated vulnerable genes (Appendix Fig. S10a-d, Details in Appendix Section 3.10).

3.1 Evaluating the predictive survival signal of the inferred SR networks

We evaluated the aggregate survival predictive signal of a pancancer SR network by testing their clinical significance in a completely independent METABRIC dataset (test set) to avoid potential risk of over-fitting. The METABRIC dataset includes the gene expression, SCNA, and survival of 1981 breast cancer patients. We first describe in this Section a general method to evaluate the capability of any SR network in predicting patient survival. Then, we present a description of survival prediction of pancancer in Section 3.2.

We define an SR pair to be *functionally active* in a tumor when its gene expression and SCNA are in *rescued* state (defined in Dataset Table E1). For an SR network if F as number of functionally *active* SR in a tumor, the predictive signal was evaluated using two complimentary approaches: (i) The predictive power of F in determining survival is estimated using a Cox regression after controlling for various confounding factor including age, sex and race. (ii) Based on F in each tumor sample, the top 10 percentile of samples were considered as *rescued* and the bottom 10 percentile as *non-rescued*. We then estimated the significance of improvement of survival in the rescued vs. non-rescued samples using a Kaplan Meier analysis.

3.2 Evaluating the predictive survival signal of pan-cancer SR networks

We studied the ability of the SRs identified to predict patient survival using the transcriptomics and copy number data of individual tumors in the independent METABERIC Breast Cancer (BC) dataset(Curtis et al,

2012). To this end, we characterized the *functional activity* of the predicted SRs in each individual tumor: A predicted DU-SR is considered to be *functionally active* in a given tumor if the vulnerable gene is down-regulated and its rescuer is up-regulated in that tumor. Analyzing the METABRIC collection, we find that tumors with many functionally active DU-SRs exhibit markedly worse patient survival than tumors with few functionally active DU-SRs, as predicted (Appendix Fig. S2i, ΔAUC = 0.20, logrank P < 4E-09, Cox-P < 7.1E-10). Similar findings hold for the DD SR types (Appendix Fig. S2k), albeit to different extent.

To illustrate that the survival prediction is not limited to breast cancer (BRCA), but also to other cancer types, we determine pancancer DU-SR survival predictive power in TCGA dataset using 2-fold cross validation. Specifically, we divided TCGA sample into two equal sets for training and testing. Applying INCISOR to training set we identified DU-SR network. Appendix Fig. S2l show fraction of SR interaction inferred (from training set) clinically significant in testing sample within each cancer types individually.

To check the robustness of the threshold used in Fig S2i (10%), we also tested thresholds: 5%,15%, and 20%. As seen in Fig S11a the AUC and logrank P-value of the KM analysis are robust with respect to the different thresholds: (i) 5% (ΔAUC=0.27 P=2.6E-06), (ii) 15% (ΔAUC=0.12 P=1.5E-06) and (iii) 20% (ΔAUC=0.17 P=1.5E-10).

3.3 Pancancer SL network and combined clinical impact of SL and SR

The functional activity of SL and SR networks determines tumor aggressiveness and patient survival. First, applying modified INCISOR (ISLE, Appendix Section 2.2) we identified SL network using pancancer TCGA samples. The survival prediction of SL is displayed in Fig S11e. We found that the clinical impact of the combined SR and SL networks is more significant than any of their individual impacts (Appendix Fig. S2j, compared with Appendix Fig. S2i) in the independent breast cancer dataset, METABERIC (Curtis et al, 2012). We assigned an SL/SR score to each patient, which combines the number of functionally active SL/SRs. We confirmed that the patients (87 samples) with both higher SL score (>90 percentile) and low SR score (<10 percentile) have significantly better survival than the patients (158 samples) with both lower SL score (<10 percentile) and high SR score (>90 percentile) (logrank p-value<6.59E-6). This combined impact is stronger than any of the single interactions.

3.4 SR network with physical protein interactions

This section summarizes synthetic rescue (SR) interactions which are mediated by human protein-protein interactions (PPI) network compiled from (Goel et al, 2012; Schaefer et al, 2012). Starting with all gene pairs with known human PPI interactions, we identified SR interactions by applying INCISOR to TCGA data. Overall, we identified 257 DU-SR interactions (with FDR < 0.1 for all INCISOR screen) mediated by direct PPI (SR-PPI); the emerging predictions are displayed in Appendix Fig. S2m (Dataset Table E6

provides the full network). The SR-PPI network was also predictive of patient's survival (Hazard Ratio =1.2, Cox $P < 4.7E-7$, $\Delta AUC = 0.17$, Appendix Fig. S2n). Surprisingly, the SR-PPI network is highly enriched with cancer driver genes (Fisher exact test $P < 6.5E-8$, Appendix Fig. S2m). And accordingly, the rescuer and vulnerable genes are highly enriched for pathways associated with cell signaling, viral carcinogenesis, estrogen signaling, PI3K-AKT etc. (Dataset Table E7,8).

3.5 DU-SR network GO enrichment

GO and KEGG enrichment analysis was conducted using R-packages *clusterprofiler (Yu et al, 2012)* and *GOFunction (Wang et al, 2011)*. The rescuer genes are enriched with ERBB/EGFR pathways, Cell cycle, Mapk and PI3k-Akt signaling pathways (full list is provided in Dataset Table E21). Processes enriched in vulnerable genes are shown in Dataset Table E22.

3.6 DU and DD SR network sparsity and characteristic

DU-SR network is a scale-free network, i.e degree distribution of SR network follows power law $A \propto d^b$ with exponential coefficient b = 1.7 (Appendix Fig. S2a). Both the degree distribution of rescuers and vulnerable gene are (Appendix Fig. S2a,b indegree exponential coefficient b = 2.1 and outdegree exponential coefficient b = 1.8).

In comparison with DU network, DD network appears to have higher density (Appendix Fig. S2c,d). This is also evident from their degree distributions. As the these networks were derived with a shared statistical tests in a symmetric manner, one may assume cautiously that this may reflect an underlying increased abundance of DD vs DR rescue interactions in cancer, but this is too early to call and further analysis is required in this regards.

3.7 SR interaction between pathways (pathway-SR network)

Next, we identified DU-SR interactions at pathway level (pathway-SR network), i.e. the genes that belong to a certain pathway are frequently rescued by the genes that belong to another pathway. The procedure involved three steps: (i) *initialization*: we find enriched pathways in all DU-SR rescuers, (ii) *vulnerable-enrichment*: for each rescuer pathways identified, we perform gene enrichment of corresponding vulnerable genes, and (iii) *rescuer-enrichment*: for each vulnerable pathway identified, we perform gene enrichment of corresponding rescuer genes. The *vulnerable-enrichment* and *rescuer-enrichment* are

repeated till convergence, which we define as 95% of vulnerable and rescuer pathways remains the same as its previous iteration. The resultant pathway-SR network is displayed in Appendix Fig. S2f.

In all the above pathway enrichment analyses we conducted both KEGG pathway enrichment using (using R package *clusterProfiler* with default parameters).

3.8 Functional similarity of DD-SR pairs

Gene ontology similarity: In order to estimate functional relationship between a rescuer and its vulnerable gene partner of DD-SR network, we used most common gene ontology *(GO) distance measure*(Yu et al, 2010), which quantifies semantic similarity between GO terms. When multiple GO terms were associated with a single gene similarity score, the maximum similarity score was taken as combined similarity score (when we change the combining method to average we obtain similar significance). We used control networks analogous to those used in case of DU-SR network (Figure S2e).
PPI and STRING distance was estimated for DD-SR network analogous to DU-SR network shown in Figure 3e.

3.9 SR events increase in tumor with advanced cancer

To study the functional activation of SRs as cancer progresses we divided the breast cancer patients in METABRIC dataset into 6 classes of cancer (removing censored data), by dividing them equally into 6 bins according to their survival times (N=627). First, in each bin, we counted the mean fraction of *functionally active* SRs. Second, we defined a vulnerable gene as *rescued* if more than N number of rescuers are over-activated with the threshold N running from 0 to 4, and counted the mean fraction of rescued vulnerable genes in the six bins.

We find that the number of SR events increases with advanced cancer stages, both in term of functionally active SRs (Appendix Fig. S2g) and mean fraction of rescued vulnerable genes (Appendix Fig. S2h), insinuating number of SR events relation with tumor resiliency.

3.10 TCGA (single nucleotide) mutation analysis

We examined the TCGA mutation profile to provide validation of SR interactions (DU-type) in pancancer-scale. (The single nucleotide polymorphism mutation profile has not been used in the SR prediction pipeline and hence can serve for independently validating INCISOR predictions.). This analysis is based on the observation that the large majority of mutations in cancer inactivate the original function of the (vulnerable) gene, and here we find that the predicted (DU-SR) rescuers of these inactivated genes indeed become

activated (via their increased expression and/or copy number alterations). If the vulnerable gene's inactivation leads to selection for rescuer activation, we expect more rescuers will be active (over-expressed and/or increased copy number) when their vulnerable partner suffers deleterious mutations. We tested this hypothesis using TCGA mutation profile that spans 5,031 patients of 23 cancer types, and we considered SR interactions of 341 genes that have mutations in at least 30 patients. Using Wilcoxon test, we statistically compared the GE and SCNA of the rescuers in patients with and without vulnerable gene mutations. The copy number of predicted DU rescuer genes is significantly higher when their vulnerable genes are mutated vs. wild type (data not used in the INCISOR inference, Wilcoxon rank-sum P <1.2E-100, cohen.d = 0.68, Appendix Fig. S10a), and so is the rescuers' gene expression (Wilcoxon rank-sum P<1.1E-17, cohen.d = 0.73). Appendix Fig. S10b shows the key vulnerable genes, when mutated, whose rescuers show significant increase both in copy number and gene-expression. Appendix Fig. S10c shows the key rescuer genes that show significant increase both in copy number and gene-expression when their vulnerable gene partners are mutated.

Our analysis revealed that CDH11, a membrane protein that mediates cell-cell adhesion and is related to ERK signaling pathways(Marie et al, 2014), is highly rescued when mutated. It was mutated in 2.1% of TCGA samples. INCISOR predicts IFT172 and MSH2 as DU rescuers of CDH11. MSH2 protein is part of mismatch repair complex (MutS), whose deregulation is associated with emergence of drug resistance. In samples where CHD11 is mutated, these rescuers show significant increase in copy number (Wilcoxon P<2.6E-6) and expression (Wilcoxon P<0.03). To investigate whether the cells are indeed functionally rescued by over-expression of rescuers genes, we examined the patients with CDH11 mutation and compared the survival of these patients when rescuers of CDH11 are highly activated to their survival when they are not. As anticipated, patients whose inactivated CHD11 is rescued show much poorer survival (Appendix Fig. S10d). This analysis demonstrates that a somatic mutation that inactivates a key cancer driver gene can be buffered/rescued by activation of rescuer genes.

3.11 Breast cancer DU and DD SR interaction

We applied INCISOR to TCGA 1098 breast cancer (BC) patient data to identify the DU-SR networks specific to breast cancer. In vitro filtering (step 1) of INCISOR was conducted using subset of in vitro screens that were performed on breast cancer cell lines. We chose breast cancer as it has the largest number of samples in the TCGA collection, and has another large cohort METABRIC(Curtis et al, 2012) on which we could test the emerging predictions in an independent manner. INCISOR identified 419 DU interactions in breast cancer (Dataset Table E32), 123 of these interactions overlap with pan cancer DU-SR

interactions. The breast cancer rescuers are enriched in EGFR tyrosine kinase inhibitor resistance, PI3K-AKT, RAS, Jak-stat and MapK signaling (Dataset Table E34). The vulnerable genes are enriched in adherens junction, proteoglycan, and carbon metabolism (Dataset Table E34). The interactions were predictive of patient survival, which we validated using Metaberic dataset (Fig S11b).

We also used breast cancer DU-SR network to predict the clinical response of 3873 patients in the TCGA dataset. Using the network and transcriptomics data of cancer patients we classified each patient to be a non-responder (or a responder) to a given drug if one or more of the rescuer partners of that drug target are over-active (and as a responder otherwise). We then compared the survival rates of the predicted responders to those of non-responders, to examine how well our predictions separated true responders and non-responders. As demonstrated, breast cancer DU-SR accurately classify patients into responder and non-responders for 15 of the drugs (Fig S11d).

We also applied INCISOR to identify DD-SR interaction specific to breast cancer. We identified 341 DD-SR interactions, 89 of these interactions were common with pancancer DD-SR interactions. The complete list of DD breast cancer specific interactions is provided in Dataset Table E33. The breast cancer DD rescuers and vulnerable genes are enriched in PI3K-AKT signaling, Human papillomavirus infection and cell cycle (Dataset Table E35). The breast cancer DD-SR interaction predict patient survival in Metaberic dataset (Fig S11c).

4 Validation of SR prediction

4.1 Benchmarking INCISOR using published SR interactions

Here, we show that INCISOR can identify with a high accuracy the molecular underpinnings of emergence of resistance to various drugs discovered by multiple extensive clinical and experimental studies. Performing literature survey of multiple clinical and experimental studies, we compiled, a large set of causal SR interactions (Methods, Dataset Table E9) that are known to determine emergence of resistance, assuming drug target of a drug as a vulnerable gene and genes causally involved in the resistance to the drug as its rescuers. Each dataset consists of experimentally validated genes whose over-expression rescues the cancer cells from a particular drug treatment in patient samples/cell lines (Datasets and associated publications are listed in Dataset Table E9, composed in total of 274 positively validated DU-SR pairs and 857 negative pairs).

The published SR dataset consists of 7 large datasets with 1132 total interactions (Dataset Table E9) for drugs ABT-737(Mills et al, 2013), BET-inhibitor(IFong et al, 2015; Rathert et al, 2015), Lapatinib(Stuhlmiller et al, 2015), Estrogen receptor inhibitor(Zhang et al, 2016), Vemurafenib (Hugo et al, 2015), Vorinostat (Falkenberg et al, 2016), BRAF inhibitor and Tamoxifen. Synthetic rescue interactions of ABT-737, Lapatinib, Estrogen receptor inhibitor and BET-inhibitors are predicted with a high accuracy with average precision >10 % at 25% recall rate (Appendix Fig. S3e-h, average AUC > 0.8, ROC curves are displayed in Appendix Fig. S3a-d, and summarized in Fig S3o). INCISOR also predicts, with moderate accuracy, the synthetic rescues in other 3 datasets (Appendix Fig. S3i-n, AUC > 0.7). Thus, INCISOR can provide a reliable set of SR pairs, which, if further validated in experiments, would have strong therapeutic impact (because they are inferred directly from patient clinical data).

To systematically evaluate the contribution of each screening step of INCISOR, we built a multivariate logistic regression based classification model, regressing the (log) p-values from the INCISOR screens. We conduct this analysis on 4 datasets, where INCISOR exhibited predictive power AUC > 0.8. Fig S3p summarizes the results obtained from the multivariate analysis, which provides an estimation of the prediction power of each INCISOR step in the overall prediction. As evident, because the contribution to the overall prediction of each screen is different across datasets, combining these screens is a good unsupervised strategy. In particular, all four screens contribute to the final prediction in INCISOR. Note that the analysis also suggests that with the availability of a larger training dataset, a supervised model could improve the accuracy of INCISOR.

4.2 *In vitro* validation of INCISOR via shRNA inhibition of DD-rescuers

Using a genome-scale shRNA screen(Marcotte et al, 2016), we validate INCISOR-predicted SR interactions in a genome-scale. Specifically, we demonstrate that when cells that have a vulnerable gene inactive due to copy number loss, a subsequent shRNA knockdown of its predicted DD-rescuer increases the cell fitness (hence show increase cell proliferation), testifying to emerging synthetic rescue in cells. The shRNA dataset composes of single knockdown of 15486 genes in 85 breast cancer cell lines(Marcotte et al, 2016). For this analysis, we removed the *in vitro-screening* from INCISOR to avoid any possible circularity. Applying INCISOR to TCGA data, we identified ***DD*** vulnerable partners of the genes that are knocked down in the breast-cancer cell line shRNA screen (N=15486) (Marcotte et al, 2016).

For each top predicted vulnerable-rescuer (V-R) gene pairs, we then classify cell lines as *conditional* cases where the V is lost (Y axis in Appendix Fig. S4h) due to copy number loss (SCNA is in bottom 10th quantile

of all cell lines for that gene); and classified the remaining cell lines as *general* cases (X axis in Appendix Fig. S4h). We predicted that the knockdown of the rescuer gene R would lead to the increase in cell growth specifically in the *conditional* cases due to DD-SR synthetic rescue effect. The *rescue-effect* for the pair is accordingly quantified as the increase in cell growth following the knockdown of the rescuer gene in *conditional* versus *general* cases. The significance of the *rescue-effect* is estimated using Wilcoxon rank sum test.

The KD of predicted DD-rescuer genes *reduces* cell growth in general across most cell lines (Appendix Fig. S4h). However, in the specific cell lines where the vulnerable partners of the DD-rescuers are lost, the knockdown of DD-rescuers *increases* cell growth, manifesting the predicted rescuing effect. Overall 65% of the predicted DD-rescuers exhibit such rescuing effects in the shRNA screen**(Marcotte et al, 2016)** (aggregate Wilcoxon P < 1.7 E-33, Cohen.d = 1.47, Methods, Appendix section 3.2)

For instance, ZNF263, a known transcriptional repressor gene, is an essential gene in cell lines (i.e, knockdown of ZNF263 in *general* case cell lines leads to decrease in cell growth, average essentiality = -0.7). However, in cell lines when its DD-vulnerable partner TAOK2 is lost, knockdown of ZNF263 promotes cellular growth (Appendix Fig. S4h inset, Wilcoxon P-value < 5.83E-05). The validation presents a large scale validation of the identified SR interactions identified by INCISOR.

4.3 *In vitro* validation of INCISOR via drug response data DD-rescuer inhibitors

Next, we provide a large-scale operative validation of the SR interactions using in vitro drug response screening data. Specifically, we show, akin to shRNA knockdown of DD-rescuers, drug inhibition of DD-rescuers results in predicted synthetic rescue in cell lines. To this end, using multiple in-vitro drug treatment compendiums(Basu et al, 2013; Cheung et al, 2011), we analyzed the anti-proliferative effect (measure in terms of IC50) of drug inhibitors of predicted DD-rescuers (Methods) in cell lines, which are known anti-tumor drugs. First, applying INCISOR to TCGA, we identified significant DD vulnerable partner genes of drug targets (rescuers) of 169 drugs in CCLE(Cheung et al, 2011)(24 drugs and 29 drug targets), and CTRP(Basu et al, 2013)(145 drugs and 185 drug targets). Using cell line SCNA, for each top predicted drug-vulnerable gene pair, we classified a cell lines exhibiting copy number loss (i.e, SCNA of the cell line falls in bottom 10% percentile all cell line and SCNA < -0.3) of the vulnerable gene as *conditional* case and all other cell lines as *general* case.

For a drug, we first convert its IC50 values across cell lines to their corresponding quantiles (qIC50). The "normalized IC50" is then defined as the mean of the qIC50 in conditional case (defined as cell line with

an inactive vulnerable gene). For example, normalized IC50=80% implies that conditional IC50 is larger than IC50 of 80% of all cell lines. This was done because the baseline IC50 of different drugs differ a lot, and the qIC50 allows to compare of the effects of vulnerable gene inactivation on the IC50 of different drugs. In the CTRP cell-line collection(Basu et al, 2013), 51% of the top 500 predicted drug-gene DD interactions show a conditional loss of effectiveness, *i.e.* IC50 was significant lower in *conditional* case as compared to *general* case (Appendix Fig. S4f, aggregate Wilcoxon P < 2.2E-16, as opposed to 0.5% significant random gene-pairs), while in the CCLE(Cheung et al, 2011) collection 35% of top predicted drug-gene pairs show such loss of effectiveness (Appendix Fig. S4g, aggregate Wilcoxon P < 2.2E-16, as opposed to 0.5% significant random gene-pairs).

In addition to the validating INCISIOR prediction, the result suggests a potential hazard of drug inhibitors of DD-rescuers, first certain anti-proliferative drugs may become ineffective or in cases may increase proliferation in patients' tumors with specific genetic background (i.e, inactive vulnerable gene); and network identified by INCISOR along with patient's tumor transcriptomic may help to proscribe specific drugs to specific patients.

4.4 *In vivo* validation of DD-SR interactions using drug response data

Next, we provide a large-scale *in vivo* validation of the DD-SR interactions using patient derived xenograft (PDX) response screening data in mice model (Gao et al, 2015). The *in vivo* drug response dataset is composed of 375 samples of mouse models of tumors treated with 38 targeted drugs totaling 2,652 patient-derived mouse xenografts (PDX) experiments (Gao et al, 2015).

Applying INCISOR to TCGA data, we identified ***DD*** vulnerable partners of gene targets of the 38 drugs.

Analogous to drug-treatment analysis (See Section 4.3), PDX were classified into *conditional* cases or *general* cases depending on whether the vulnerable gene is lost or not for each top predicted vulnerable-rescuer gene pair. We predicted that a drug inhibiting of the rescuer gene would lead to an increase in tumor growth in the *conditional* cases due to DD synthetic rescue effect. Indeed, pharmacological inhibition of predicted DD-rescuer genes show marked increase in tumor proliferation in the particular PDX where the partnered vulnerable genes are lost (*conditional* case), both regarding mean (Wilcoxon P-value < 2.2E-16, Appendix Fig. S4d) and maximum (Appendix Fig. S4c, Wilcoxon P-value < 2.2E-16) tumor size reduction, affirming the predicted rescues. The *rescue-effect* for the pair is quantified as the difference of tumor growth following the treatment of the drug inhibitor of rescuer gene in *conditional* versus *general* cases.

The analysis suggests that drug treatments targeting genes whose DD vulnerable partner genes may be down-regulated in specific tumors, may be counter-productive and in cases hazardous. To showcase this, we compare between *conditional* and *general* cases progression free survival (PFS) of mice xenograft treated with the 36 drugs. As illustrated in Appendix Fig. S4e *conditional* xenografts exhibits 49.7 % decrease in mean PFS as compared to *general* xenografts.

4.5 Re-sensitizing patient-derived resistant cell lines to ALK/EGFR inhibitor

We analyzed a large-scale drug screen identifying drugs sensitizing patient-derived cell lines from tumors resistant to ALK and EGFR inhibitors (Crystal et al, 2014). We conjectured that a drug targeting a predicted DU rescuer of ALK (or EGFR) would successfully sensitize the corresponding treatment-resistant cells. Indeed, we find that the strength of the DU-SR interaction between the drugs' targets and ALK/EGFR is significantly correlated with the observed *in vitro* efficiency of the drugs tested (Fig S4a). Moreover, INCISOR accurately identifies top 2% drugs that re-sensitize patient-derived resistant cell lines to treatment with EGFR and ALK inhibitors (AUC = .82, Appendix Fig. S4b, Methods), evaluated by a standard ROC analysis.

Conducting a large-scale *in vitro* drug screen experiment on cell lines derived from the tumors of EGFR-resistant patients and ALK-resistant patients, Crystal et. al. (Crystal et al, 2014) experimentally determined the potential of each drug to sensitize the tumors back to the EGFR or ALK therapy. To predict sensitization potential of a screened drug, we estimate the strength of DU-SR interactions (*interaction-score;* described above) between the targets of the drug and EGFR/ALK using INCISOR. The maximum of the drug targets *interaction-scores* was estimated to be the drug's sensitization potential. Fig S4a compares the INCISOR-predicted and experimentally determined sensitization potentials for the drugs screened.

4.6 Experimental validation of DD-SR in head and neck cancer cell lines

We conducted a large-scale double gene knockdown experiment combining in vitro shRNA knockdowns (KDs) and drug treatments (Appendix Fig. S5a) to evaluate the percentage of (DD) SR interactions predicted by INCISOR that exhibit significant rescue effects. To test our ability to predict and experimentally validate predicted SR interaction of a key rescuer gene, we studied the role of mTOR as a predicted rescuer gene in head and neck squamous cell carcinoma (HNSC), where is it thought to play an important role(Iglesias-Bartolome et al, 2013). Rapamycin specifically targets mTOR in HNSC(Amornphimoltham et al, 2008; Iglesias-Bartolome et al, 2013; Yamaguchi et al, 2016) (particularly in the HN12 cell line we used in our experiment). Specifically, Rapamycin targets mTOR complex1 indirectly by binding to FKBP12 gene(Amornphimoltham et al, 2008; Iglesias-Bartolome et al, 2013; Yamaguchi et al, 2016). And hence the

rapamycin enables to target a predicted rescuer gene by a highly specific drug, combined with the ability to knock down predicted vulnerable genes in a clinically-relevant lab setting. To this end we studied DD-SR predictions in a HNSC cell-line HN12, which, like most HNSC cells, is highly sensitive to rapamycin(Amornphimoltham et al, 2005). Detailed information on the shRNA sequence and cell counts are listed in Dataset Table E10.

Appendix Fig. S5a summarizes the overall experimental procedure. After which mTOR was inactivated via Rapamycin treatment, 2200 genes were knocked down by pooled shRNA in HN12 cell lines. HN12 cells were infected with a library of retroviral barcoded shRNAs at a representation of ~1,000 and a multiplicity of infection (MOI) of ~0.3, including at least 2 independent shRNAs for each gene of interest and controls. At day 3 post infection cells were selected with puromycin for 3 days (1µg/ml) to remove the minority of uninfected cells. After that, cells where expanded in culture for 3 days and then an initial population-doubling 0 (PD0) sample was taken. For *in vitro* testing, the cells were divided into 6 populations, 3 were kept as a control and 3 where treated with rapamycin (100nM). Cells where propagated in the presence or not of drug for an additional 12 doublings before the final, PD13 sample was taken. shRNA barcode was PCR-recovered from genomic samples and samples sequenced to calculate abundance of the different shRNA probes. From these shRNA experiments, we obtained cell counts for each gene knock-down at the following three time points: (a) post shRNA infection (PD0, referred as *initial count*), (b) shRNA treatment followed by either Rapamycin treatment (PD13, referred as *treated count,* 3 replicates) or control (PD13, referred as *untreated count,* 3 replicates) (c) shRNA infected cell injected to mice (tumor, referred as *in-vivo count*, 2 replicates). To obtain *normalized* counts at each time point, cell counts of each shRNA at each time point were divided by corresponding total number of cell count.

Appendix Fig. S5a shows overall experimental design of the pooled-shRNA+Drug treatment experiment. The rescue effect of mTOR inhibition was quantified as the observed increase in cell growth post rapamycin-treatment + knockdown relative to the cell growth observed after the knockdown without rapamycin treatment. Fig S5b show rescue effect of top 11 vulnerable partner of mTOR due to rapamycin treatment. Appendix Fig. S5c shows the cellular viability (in terms of cell count) of these genes following the rescuer-shRNA and rescuer-shRNA + rapamycin treatment for each gene KD(shRNAs of same gene were combined). Appendix Fig. S5c (measurements per gene) and S5d (measurements per shRNA level) display the cellular viability post-rapamycin treatment with and without shRNA.

4.7 Experimental validation of SR-based combinational therapies in head and neck cancer

4.7.1 Validating synergism of SR based drug combinations

Each of drug combination for multiple concentration combination and was carried in 3 technical replicates. Raw data is provided in Dataset Table E11-17 and Appendix Fig. S6, for each of 7 drug-combinations, dose matrix combinatorial drug treatment (48h) in 5 HNSC cell lines (Cal27, Cal33, HN12, Detroit 562, HN12 and SCC147). Numbers in the matrix represents percentage cell viability following drug treatment (mean viability of the three replicates). Appendix Fig. S7 shows show Fa-CI curves created based on the matrix data by using dose ratios for each combination are indicated. The Y axis displays the combination index (CI; synergism CI < 1, additivity effect CI = 1, antagonism CI > 1) at different levels of growth inhibition (Fraction affected, X axis). Combination index for quantitation of drug synergy was constructed by CompuSyn software(Chou, 2006; Chou, 2010). CI values represent synergism (CI<1), additivity (CI=1), and antagonism (CI>1), respectively.

Appendix Fig. S5e and S5f are representative dose matrix combinatorial treatment and FA-CI curve shown for a representative predicted combination (Dasatinib + BYL719) in a representative cell line (HN12). Similar analysis was conducted to for each combinations and each cell lines tested (Fig S6, S7) to generate **Figure 2b**.

4.7.2 Validating synergism of SR based drug combinations via siRNA experiments

We tested the re-sensitization of treatment with BYL719 by siRNA knockdowns of its predicted rescuer gene mTOR in four cell lines (Cal33, HN12, Detroit 562, and SCC147; Appendix Fig. S8i, left four columns; Appendix Fig. S8e-h; Dataset Table E18) and similarly, the re-sensitization of treatment with Dasatinib by siRNA knockdowns of its predicted rescuer PIK3CA (Appendix Fig. S8i, right four columns; Appendix Fig. S8a-d, Dataset Table E19). As evident, these knockdowns significantly enhance the cell lines sensitivity to the primary drugs in all 4 of cell lines tested, as predicted (Appendix Fig. S8i). We also further validate the knock down efficiency of the siRNA library used in prior studies (Lee et al, 2014), which were used in the combinatorial drug evaluation analysis. These results are now reported in Figure S11h. Appendix Fig. S8a-h show drug response curves of Dasatinib (or BYL719) in cell lines following the treatment of rescuer or control siRNA, constructed using DRC package.

5 Applications of DU-SR network

5.1 Drug-specific SR network

Appendix Fig. S10e display the DU-SR of 28 targeted drugs (Methods). The triangles in figure display targeted drugs. Red circle are vulnerable gene, green circles show predicted DU rescuer genes.

5.2 Tumor relapse prediction in ovarian and breast cancer

Beyond initial drug response, our overarching hypothesis suggests that SR circuits might contribute to adaptive evolution in tumors after a drug insult, and thus to tumor relapse. To test this, we analyzed **(a)** longitudinal expression and sequencing data of 81 stage-II, III ovarian cancer patients (OC81 dataset) treated with Taxane(Patch et al, 2015); and **(b)** 155 primary breast cancer patients treated with Tamoxifen(Chanrion et al, 2008).

To test the utility of SRs in predicting the emergence of resistance to cancer therapy we analyzed the expression data from tumors of 81 ovarian cancer patients. The patients had been treated with Taxane, which has 14 predicted DU rescuer genes linked to 3 drug targets in the treatment specific DU-SR network(Patch et al, 2015). Six out of these genes were significantly over-expressed in pretreatment tumor data of non-responder versus responder patients (Wilcoxon rank-sum $P<1.5E-4$). Ten out of the 14 predicted rescuers had increased rescuers' activation in tumors of patients that initially responded but then relapsed (overall Wilcoxon rank-sum $P< 5E-32$, Appendix Fig. S10f). Among those are some previously known modulators of resistance to taxane including TPX2(Warner et al, 2009), EREG(Galletti et al, 2014), and SLCO1B3(de Morree et al, 2016).

Among them the TPX2 gene has been shown to be essential for resistance emergence to taxane and its knockdown is shown to be re-sensitize the taxane-resistant-tumors to taxane treatment(Warner et al, 2009). The over-expression of EREG(Galletti et al, 2014) or SLCO1B3(de Morree et al, 2016) have also been independently shown to reduce sensitivity to Taxane therapy.

We further analyzed the expression data of 155 primary breast cancer patients who were treated with Tamoxifen(Chanrion et al, 2008), where tumor relapsed in 52 patients within 5 years. With the activity states of 4 rescuers of Tamoxifen's drug targets, we built a SVM classifier to predict if patient will relapse or not based on gene expression from their tumor. The binary classifier was able to accurately predict the patients whose tumor will remerge (fivefold cross-validation AUC=0.74, Appendix Fig. S10g).

5.3 INCISOR successfully identifies drugs re-sensitizing resistant cell lines to the primary cancer drugs

We present a large-scale validation that inhibition of DU rescuer of target of a drug re-sensitize the cell line to the drug using a large-scale *in vitro* drug combination dataset spanning all combinations of 108 drugs in

melanoma cell-lines (approximately 6000 drug combinations tested)(Friedman et al, 2015). For each drug, we first identified the cell-lines resistant to it. Second, we conjectured that cells resistant to the drug would be sensitized by inhibition of its (DU) rescuer gene, implying that drug combination of the drug and inhibitor of its DU rescuers will be synergistic in resistant cell lines. Indeed we find that the pharmacological inhibition of these predicted DU-rescuers are synergistic (AUC > .86, Appendix Fig. S9a,b). Technical description of analyses is provided below.

We first compiled a positive set of synergistic combination of primary and secondary drugs in the resistant cell lines. For each primary drug treatment, we first identified cell lines resistant to it. A cell line was considered resistant to the drug if its individual treatment does not decrease cellular viability in the cell line. Next, we identify secondary drugs that exhibit significant synergism with the primary drug in the resistant cell lines. The significance of the synergism is determined as follows.

We used a novel method to call the significance of synergism from the screen, which shows better consistency in the data and drastically reduces false positives compared to the simple application of the Bliss formula (Amzallag et al, submitted). Briefly, the measured cell viability was median polished to avoid bias linked to the rows and columns of each plate (Mosteller et al, 1983). Then, for each cell line screen at a given fixed drug dose, we fit a linear model to the combination viabilities based on Bliss Independence assumption (Bliss, 1939). We recomputed the singlet viabilitites and estimated their standard error. We then generated a null hypothesis and estimated synergism as the deviance from the Bliss independence (i.e. the residuals of the linear system). We estimated the noise in the data using the variance on the DMSO wells on each plate and the squared standard error of the estimated singlets. The P value represents as the likelihood of a residual's absolute value of being greater than observed given the null hypothesis of independent drug effects and the noise in the data. We corrected the P values for FDR with the Benjamini-Hochberg procedure and called synergism combinations with P values less than 0.05 and positive Z values, that is, combinations with viability significantly lower than the null hypothesis combination viability. The manuscript describing the statistical estimation of the drug synergism from the experiment is currently under review (Amzallag et al, submitted).

We determine SR ability to accurately predict the positive set of synergetic combinations. For each drug combination screened (Friedman et al, 2015), we estimated strength of DU-SR interaction (*interaction-scores*) of all drug-target pairings of the combination and used the maximum of *interaction-scores* as a prediction of its synergism. Finally, we evaluated the accuracy of estimated synergism to predict the positive set using ROC analysis and prediction-recall statistics (Appendix Fig. S9a,b).

5.4 Identification of in vivo synergistic drug combinations involving Benemitib (MEKi)

INCISOR successfully predicts synergistic treatments *in vivo* in a patient-derived mouse xenografts (PDX) dataset (Gao et al, 2015). We studied eight drugs that were combined with the MEK inhibitor Binimetinib (Waterfall et al, 2014) in 375 mouse models, with 1103 xenografts with individual drug treatment and 930 xenografts with one of the combinations 8 drug with Binimetinib. Among 8 combinations three target predicted DU and one targets predicted DD rescuer of MEK (Dataset Table E20).

For testing SR-based synergistic combination *in vivo*, we analyzed a collection of in vivo mouse xenograft models that cover eight drug combinations involving, MEK inhibitor, Benemitib in 375. For each predicted rescuer gene, we compared progression-free survival of PDXs treated with (i) Benmitinib alone, (ii) a drug inhibitor of the rescuer alone and (iii) the drug combination of both (i) and (ii).

Remarkably, compared with PDXs treated with Binimetinib alone, all three predicted DU-SR combination treatments increased progression-free survival in the PDXs (Figure S9c-e), while the predicted DD-SR combination decreased progression-free survival, as expected (Appendix Fig. Sf). This point out the targeting DU rescuer are synergistic while targeting DD inhibitors are antagonistic, suggesting that predicted sensitizations translate to actual *in-vivo* survival benefits in mice model.

5.5 SR based supervised prediction of resistance to immune checkpoint blockades

We analyzed three independent datasets to show role of SR in immunotherapy (Figure 4a):

5.5.1 Hugo et al. **(Hugo et al, 2016)**

The dataset composed of 37 metastatic melanoma patients treated with anti-PD1 therapy. We first predicted DU and DD rescuers of PD1/PDL1 by applying INCISOR to TCGA dataset. Using expression of these rescuers as features, we built an SVM based supervised model. We conducted 5 fold cross-validation to determine the accuracy of the predictor in predicting resistance to anti-PD1. Feature selection was conducted on predicted rescuers using half of the training data. The other half of training data was used to train the model. The test data used to determine accuracy was neither used for feature selection nor training to avoid any over-fitting.

5.5.2 Prat et al. (Prat et al, 2017)

The dataset composed of 65 patients with melanoma, lung non-squamous, squamous cell lung or head and neck cancer treated with anti-PD1 therapy. The pre-treatment transcriptome was conducted using Nanostring nCounter technology in the dataset. Analogous to Hugo et al. dataset analysis, we built a supervised predictor for the dataset by training an SVM using rescuers of PD1/PDL1 as features. The accuracy of the predictor was estimated using 5 fold cross-validation.

5.5.3 Van Allen et al. (Van Allen et al, 2015)

The dataset composed of 40 metastatic melanoma patients treated with anti-CLTA4. An SVM was trained using the pre-treatment expression of rescuers of CTLA4 as features. Analogous to the previous analysis described above, feature selection was conducted before training the SVM. The accuracy of predictor was estimated using 5 fold cross-validation. The test data used to determine the accuracy neither used for training nor for feature selection to avoid any overfitting.

5.6 Using SR strength to predict drug response in TCGA

We checked if using the strength of SR improves the prediction. Specifically, we weight the functionally active rescuers by the SR strength, i.e, the SR score assigned by INCISOR. As shown in Fig S11g, this strategy shows significant predictive power for 13 drugs. compared to 14 drugs whose response can be predicted using the unweighted SR counts (the number of upregulated rescuer).

6 SR based therapeutics opportunities

The functional activity of SL and SR networks determines tumor aggressiveness and patient survival. We demonstrate here that the clinical impact of the combined SR and SL networks is more significant than their individual impacts (Appendix Fig. S2j). The SL network provides information on the selectivity and efficacy of a given drug(Jerby-Arnon et al, 2014). As pointed out above, the SR network provides complementary information on the likelihood to incur resistance. Combining SL and SR networks, we can predict a drug that has the highest efficacy/selectivity and lowest chance of developing resistance.

SR reprogramming can be used to develop two novel classes of sequential treatment regimens of anticancer therapies. First, almost all cancer patients who initially responded to a drug, have the potential to develop resistance to the treatment and experience tumor relapse. Currently, we do not have the ability to access and prepare for the second line of treatment for the relapsed tumors, till it happens to the patients, which is often too late. SR provides a way to infer, together with pretreatment expression screening, whether resistance

will emerge quickly and, more importantly, the possible mechanisms of the emergence of resistance and how they can be mitigated by subsequent treatments. Therefore, SR can guide decisions on the second line of action without biopsies from the relapsed tumors.

Second, some of the targeted anti-cancer therapies are known to be more efficient and effective in treating cancer (eg. kinase inhibitors) than other drugs, provided tumors are homogenously addicted to their target gene. Using SR interaction between the target gene (as rescuer) and its vulnerable partners, it is possible to make the tumor population homogeneous by targeting the vulnerable partners of the rescuer. In response to the vulnerable gene inactivation, cancer cells will over-activate the rescuer, which will lead to oncogenic (or non-oncogenic) addiction(Weinstein, 2002). In the second line of treatment, the rescuer can be targeted to eradicate the homogeneous tumor population, thus efficiently treating cancer.

7 Limitation of INCISOR

INCISOR has limitations arising from the scarcity of available data, the specific design of the pipeline, and the diverse mechanisms of the emergence of drug resistance.

It is well-known that many genes are correlated based on their expression and the proximal genes have correlated SCNA values, which make it difficult to identify the true rescuers from spurious ones. INCISOR mitigates some of these problems by selecting pairs only when they are supported by both gene expression and SCNA. Also, combination of shRNA and phylogenetic screening are less prone to this confounder. INCISOR is also based on patient survival data, which is known to be noisy. INCISOR does not incorporate other genetic, epigenetic and post-transcriptional mechanism of gene inactivation partly due to the unavailability of these data for cancer patients.

INCISOR is designed to identify the rescuer genes for targeted therapies, so it cannot be used to predict drug response/resistance analysis for non-targeted therapies such as generic chemotherapy (e.g. Cisplatin). By definition, SR reprogramming events are context-specific to a cancer type or a sub-type. Our pancancer SR network focuses on the generic SR interactions that are prevalent across multiple cancer types, and the same pipeline can be applied to specific cancer types or sub-types as presented in the main text and Appendix for specific cancer types and subtypes.

It must be noted that resistance does not always emerge due to SR reprogramming. This is because there are multiple mechanisms for development of resistance including drug efflux via multi-drug resistance mechanism or the modification of drug target that makes drug ineffective. We nonetheless note that SR interactions are so widespread in multiple cancers that they are highly likely to be a contributing factor. Our

analysis shows that only a small subset of SR interactions is mediated by physical contacts, and further studies are needed to identify the mechanism of SR reprogramming in giving rise to drug resistance.

The in-vitro and in-vivo evidence supports a future role of SRs in devising anti-resistant combination therapies. However, further experimental and clinical studies will be obviously required to study this further.

We expect the fast growth of the publically available omics/survival patient data, both within the TCGA collection and beyond would help us designing a better pipeline and improving our identification of the SR interactions, and lead to a deeper understanding of their mechanism in a context-specific manner.

8 Reference:

Amornphimoltham P, Patel V, Leelahavanichkul K, Abraham RT, Gutkind JS (2008) A retroinhibition approach reveals a tumor cell-autonomous response to rapamycin in head and neck cancer. *Cancer Research* **68:** 1144-1153

Amornphimoltham P, Patel V, Sodhi A, Nikitakis NG, Sauk JJ, Sausville EA, Molinolo AA, Gutkind JS (2005) Mammalian target of rapamycin, a molecular target in squamous cell carcinomas of the head and neck. *Cancer Res* **65:** 9953-9961

Amzallag A, Pruteanu-Malinici J, Friedman AA, Haber DA, Ramaswamy S, Fisher DE, Benes CH (submitted) Statistical Assessment of Synergy in a Large Replicate-less Drug Combination Screen.

Basu A, Bodycombe NE, Cheah JH, Price EV, Liu K, Schaefer GI, Ebright RY, Stewart ML, Ito D, Wang S, Bracha AL, Liefeld T, Wawer M, Gilbert JC, Wilson AJ, Stransky N, Kryukov GV, Dancik V, Barretina J, Garraway LA et al (2013) An interactive resource to identify cancer genetic and lineage dependencies targeted by small molecules. *Cell* **154:** 1151-1161

Bliss CI (1939) The toxicity of poisons applied jointly. *Annals of Applied Biology* **26:** 585-615

Chanrion M, Negre V, Fontaine H, Salvetat N, Bibeau F, Mac Grogan G, Mauriac L, Katsaros D, Molina F, Theillet C, Darbon JM (2008) A gene expression signature that can predict the recurrence of tamoxifen-treated primary breast cancer. *Clin Cancer Res* **14:** 1744-1752

Cheung HW, Cowley GS, Weir BA, Boehm JS, Rusin S, Scott JA, East A, Ali LD, Lizotte PH, Wong TC, Jiang G, Hsiao J, Mermel CH, Getz G, Barretina J, Gopal S, Tamayo P, Gould J, Tsherniak A, Stransky N et al (2011) Systematic investigation of genetic vulnerabilities across cancer cell lines reveals lineage-specific dependencies in ovarian cancer. *Proc Natl Acad Sci U S A* **108:** 12372-12377

Chou TC (2006) Theoretical basis, experimental design, and computerized simulation of synergism and antagonism in drug combination studies. *Pharmacol Rev* **58:** 621-681

Chou TC (2010) Drug combination studies and their synergy quantification using the Chou-Talalay method. *Cancer Res* **70:** 440-446

Cowley GS, Weir BA, Vazquez F, Tamayo P, Scott JA, Rusin S, East-Seletsky A, Ali LD, Gerath WF, Pantel SE, Lizotte PH, Jiang G, Hsiao J, Tsherniak A, Dwinell E, Aoyama S, Okamoto M, Harrington W, Gelfand E, Green TM et al (2014) Parallel genome-scale loss of function screens in 216 cancer cell lines for the identification of context-specific genetic dependencies. *Sci Data* **1:** 140035

Crystal AS, Shaw AT, Sequist LV, Friboulet L, Niederst MJ, Lockerman EL, Frias RL, Gainor JF, Amzallag A, Greninger P, Lee D, Kalsy A, Gomez-Caraballo M, Elamine L, Howe E, Hur W, Lifshits E, Robinson HE, Katayama R, Faber AC et al (2014) Patient-derived models of acquired resistance can identify effective drug combinations for cancer. *Science* **346:** 1480-1486

Curtis C, Shah SP, Chin S-F, Turashvili G, Rueda OM, Dunning MJ, Speed D, Lynch AG, Samarajiwa S, Yuan Y, Gräf S, Ha G, Haffari G, Bashashati A, Russell R, McKinney S, Caldas C, Aparicio S, Curtis† C, Shah SP et al (2012) The genomic and transcriptomic architecture of 2,000 breast tumours reveals novel subgroups. *Nature*

de Morree ES, Bottcher R, van Soest RJ, Aghai A, de Ridder CM, Gibson AA, Mathijssen RH, Burger H, Wiemer EA, Sparreboom A, de Wit R, van Weerden WM (2016) Loss of SLCO1B3 drives taxane resistance in prostate cancer. *Br J Cancer* **115:** 674-681

Falkenberg KJ, Newbold A, Gould CM, Luu J, Trapani JA, Matthews GM, Simpson KJ, Johnstone RW (2016) A genome scale RNAi screen identifies GLI1 as a novel gene regulating vorinostat sensitivity. *Cell Death Differ* **23:** 1209-1218

Friedman AA, Amzallag A, Pruteanu-Malinici I, Baniya S, Cooper ZA, Piris A, Hargreaves L, Igras V, Frederick DT, Lawrence DP, Haber DA, Flaherty KT, Wargo JA, Ramaswamy S, Benes CH, Fisher DE (2015) Landscape of Targeted Anti-Cancer Drug Synergies in Melanoma Identifies a Novel BRAF-VEGFR/PDGFR Combination Treatment. *PLoS One* **10:** e0140310

Galletti G, Matov A, Beltran H, Fontugne J, Miguel Mosquera J, Cheung C, MacDonald TY, Sung M, O'Toole S, Kench JG, Suk Chae S, Kimovski D, Tagawa ST, Nanus DM, Rubin MA, Horvath LG, Giannakakou P, Rickman DS (2014) ERG induces taxane resistance in castration-resistant prostate cancer. *Nat Commun* **5:** 5548

Gao H, Korn JM, Ferretti S, Monahan JE, Wang Y, Singh M, Zhang C, Schnell C, Yang G, Zhang Y, Balbin OA, Barbe S, Cai H, Casey F, Chatterjee S, Chiang DY, Chuai S, Cogan SM, Collins SD, Dammassa E et al (2015) High-throughput screening using patient-derived tumor xenografts to predict clinical trial drug response. *Nat Med* **21:** 1318-1325

Goel R, Harsha HC, Pandey A, Prasad TS (2012) Human Protein Reference Database and Human Proteinpedia as resources for phosphoproteome analysis. *Mol Biosyst* **8:** 453-463

Gonzalez-Malerva L, Park J, Zou L, Hu Y, Moradpour Z, Pearlberg J, Sawyer J, Stevens H, Harlow E, LaBaer J (2011) High-throughput ectopic expression screen for tamoxifen resistance identifies an atypical kinase that blocks autophagy. *Proc Natl Acad Sci U S A* **108:** 2058-2063

Hugo W, Shi HB, Sun L, Piva M, Song CY, Kong XJ, Moriceau G, Hong AY, Dahlman KB, Johnson DB, Sosman JA, Ribas A, Lo RS (2015) Non-genomic and Immune Evolution of Melanoma Acquiring MAPKi Resistance. *Cell* **162:** 1271-1285

Hugo W, Zaretsky JM, Sun L, Song C, Moreno BH, Hu-Lieskovan S, Berent-Maoz B, Pang J, Chmielowski B, Cherry G, Seja E, Lomeli S, Kong X, Kelley MC, Sosman JA, Johnson DB, Ribas A, Lo RS (2016) Genomic and Transcriptomic Features of Response to Anti-PD-1 Therapy in Metastatic Melanoma. *Cell* **165:** 35-44

IFong CY, Gilan O, Lam EY, Rubin AF, Ftouni S, Tyler D, Stanley K, Sinha D, Yeh P, Morison J, Giotopoulos G, Lugo D, Jeffrey P, Lee SC, Carpenter C, Gregory R, Ramsay RG, Lane SW, Abdel-Wahab O, Kouzarides T et al (2015) BET inhibitor resistance emerges from leukaemia stem cells. *Nature* **525:** 538-542

Iglesias-Bartolome R, Martin D, Gutkind JS (2013) Exploiting the head and neck cancer oncogenome: widespread PI3K-mTOR pathway alterations and novel molecular targets. *Cancer Discov* **3:** 722-725

Jerby-Arnon L, Pfetzer N, Waldman YY, McGarry L, James D, Shanks E, Seashore-Ludlow B, Weinstock A, Geiger T, Clemons PA, Gottlieb E, Ruppin E (2014) Predicting cancer-specific vulnerability via data-driven detection of synthetic lethality. *Cell* **158:** 1199-1209

Lee JS, Das A, Jerby-Arnon L, Arafeh R, Auslander N, Davidson M, McGarry L, James D, Amzallag A, Park SG, Cheng K, Robinson W, Atias D, Stossel C, Buzhor E, Stein G, Waterfall JJ, Meltzer PS, Golan T, Hannenhalli S et al (2018) Harnessing synthetic lethality to predict the response to cancer treatment. *Nat Commun* **9:** 2546

Lee LC, Gao S, Li Q, Luo J (2014) Using pooled miR30-shRNA library for cancer lethal and synthetic lethal screens. *Methods Mol Biol* **1176:** 45-58

Marcotte R, Brown KR, Suarez F, Sayad A, Karamboulas K, Krzyzanowski PM, Sircoulomb F, Medrano M, Fedyshyn Y, Koh JL, van Dyk D, Fedyshyn B, Luhova M, Brito GC, Vizeacoumar FJ, Vizeacoumar FS, Datti A, Kasimer D, Buzina A, Mero P et al (2012) Essential gene profiles in breast, pancreatic, and ovarian cancer cells. *Cancer Discov* **2:** 172-189

Marcotte R, Sayad A, Brown KR, Sanchez-Garcia F, Reimand J, Haider M, Virtanen C, Bradner JE, Bader GD, Mills GB, Pe'er D, Moffat J, Neel BG (2016) Functional Genomic Landscape of Human Breast Cancer Drivers, Vulnerabilities, and Resistance. *Cell* **164:** 293-309

Marie PJ, Hay E, Modrowski D, Revollo L, Mbalaviele G, Civitelli R (2014) Cadherin-mediated cell-cell adhesion and signaling in the skeleton. *Calcif Tissue Int* **94:** 46-54

Mills JR, Malina A, Lee T, Di Paola D, Larsson O, Miething C, Grosse F, Tang H, Zannis-Hadjopoulos M, Lowe SW, Pelletier J (2013) RNAi screening uncovers Dhx9 as a modifier of ABT-737 resistance in an Eμ-myc/Bcl-2 mouse model. *Blood* **121:** 3402-3412

Mosteller F, Tukey JW, Hoaglin DC (1983) *Understanding robust and exploratory data analysis*, New York: Wiley.

Patch A-M, Christie EL, Etemadmoghadam D, Garsed DW, George J, Fereday S, Nones K, Cowin P, Alsop K, Bailey PJ, Kassahn KS, Newell F, Quinn MCJ, Kazakoff S, Quek K, Wilhelm-Benartzi C, Curry E, Leong HS,

Hamilton A, Mileshkin L et al (2015) Whole–genome characterization of chemoresistant ovarian cancer. *Nature* **521:** 489-494

Prat A, Navarro A, Pare L, Reguart N, Galvan P, Pascual T, Martinez A, Nuciforo P, Comerma L, Alos L, Pardo N, Cedres S, Fan C, Parker JS, Gaba L, Victoria I, Vinolas N, Vivancos A, Arance A, Felip E (2017) Immune-Related Gene Expression Profiling After PD-1 Blockade in Non-Small Cell Lung Carcinoma, Head and Neck Squamous Cell Carcinoma, and Melanoma. *Cancer Res* **77:** 3540-3550

Rathert P, Roth M, Neumann T, Muerdter F, Roe J-S, Muhar M, Deswal S, Cerny-Reiterer S, Peter B, Jude J, Hoffmann T, Boryń ŁM, Axelsson E, Schweifer N, Tontsch-Grunt U, Dow LE, Gianni D, Pearson M, Valent P, Stark A et al (2015) Transcriptional plasticity promotes primary and acquired resistance to BET inhibition. *Nature* **525:** 543-547

Schaefer MH, Fontaine JF, Vinayagam A, Porras P, Wanker EE, Andrade-Navarro MA (2012) HIPPIE: Integrating protein interaction networks with experiment based quality scores. *PLoS One* **7:** e31826

Stuhlmiller TJ, Miller SM, Zawistowski JS, Nakamura K, Beltran AS, Duncan JS, Angus SP, Collins KA, Granger DA, Reuther RA, Graves LM, Gomez SM, Kuan PF, Parker JS, Chen X, Sciaky N, Carey LA, Earp HS, Jin J, Johnson GL (2015) Inhibition of Lapatinib-Induced Kinome Reprogramming in ERBB2-Positive Breast Cancer by Targeting BET Family Bromodomains. *Cell Rep* **11:** 390-404

Van Allen EM, Miao D, Schilling B, Shukla SA, Blank C, Zimmer L, Sucker A, Hillen U, Foppen MHG, Goldinger SM, Utikal J, Hassel JC, Weide B, Kaehler KC, Loquai C, Mohr P, Gutzmer R, Dummer R, Gabriel S, Wu CJ et al (2015) Genomic correlates of response to CTLA-4 blockade in metastatic melanoma. *Science* **350:** 207-211

Wang J, Zhou X, Zhu J, Gu Y, Zhao W, Zou J, Guo Z (2011) GO-function: deriving biologically relevant functions from statistically significant functions. *Briefings in bioinformatics* **13:** 216-227

Warner SL, Stephens BJ, Nwokenkwo S, Hostetter G, Sugeng A, Hidalgo M, Trent JM, Han H, Von Hoff DD (2009) Validation of TPX2 as a potential therapeutic target in pancreatic cancer cells. *Clin Cancer Res* **15:** 6519-6528

Waterfall JJ, Arons E, Walker RL, Pineda M, Roth L, Killian JK, Abaan OD, Davis SR, Kreitman RJ, Meltzer PS (2014) High prevalence of MAP2K1 mutations in variant and IGHV4-34-expressing hairy-cell leukemias. *Nat Genet* **46:** 8-10

Weinstein IB (2002) Cancer. Addiction to oncogenes--the Achilles heal of cancer. *Science* **297:** 63-64

Yamaguchi K, Iglesias-Bartolome R, Wang Z, Callejas-Valera JL, Amornphimoltham P, Molinolo AA, Cohen EE, Califano JA, Lippman SM, Luo J, Gutkind JS (2016) A synthetic-lethality RNAi screen reveals an ERK-mTOR co-targeting pro-apoptotic switch in PIK3CA+ oral cancers. *Oncotarget* **7:** 10696-10709

Yu G, Li F, Qin Y, Bo X, Wu Y, Wang S (2010) GOSemSim: an R package for measuring semantic similarity among GO terms and gene products. *Bioinformatics* **26:** 976-978

Yu G, Wang L-G, Han Y, He Q-Y (2012) clusterProfiler: an R package for comparing biological themes among gene clusters. *Omics: a journal of integrative biology* **16:** 284-287

Zhang YW, Nasto RE, Varghese R, Jablonski SA, Serebriiskii IG, Surana R, Calvert VS, Bebu I, Murray J, Jin L, Johnson M, Riggins R, Ressom H, Petricoin E, Clarke R, Golemis EA, Weiner LM (2016) Acquisition of estrogen independence induces TOB1-related mechanisms supporting breast cancer cell proliferation. *Oncogene* **35:** 1643-1656

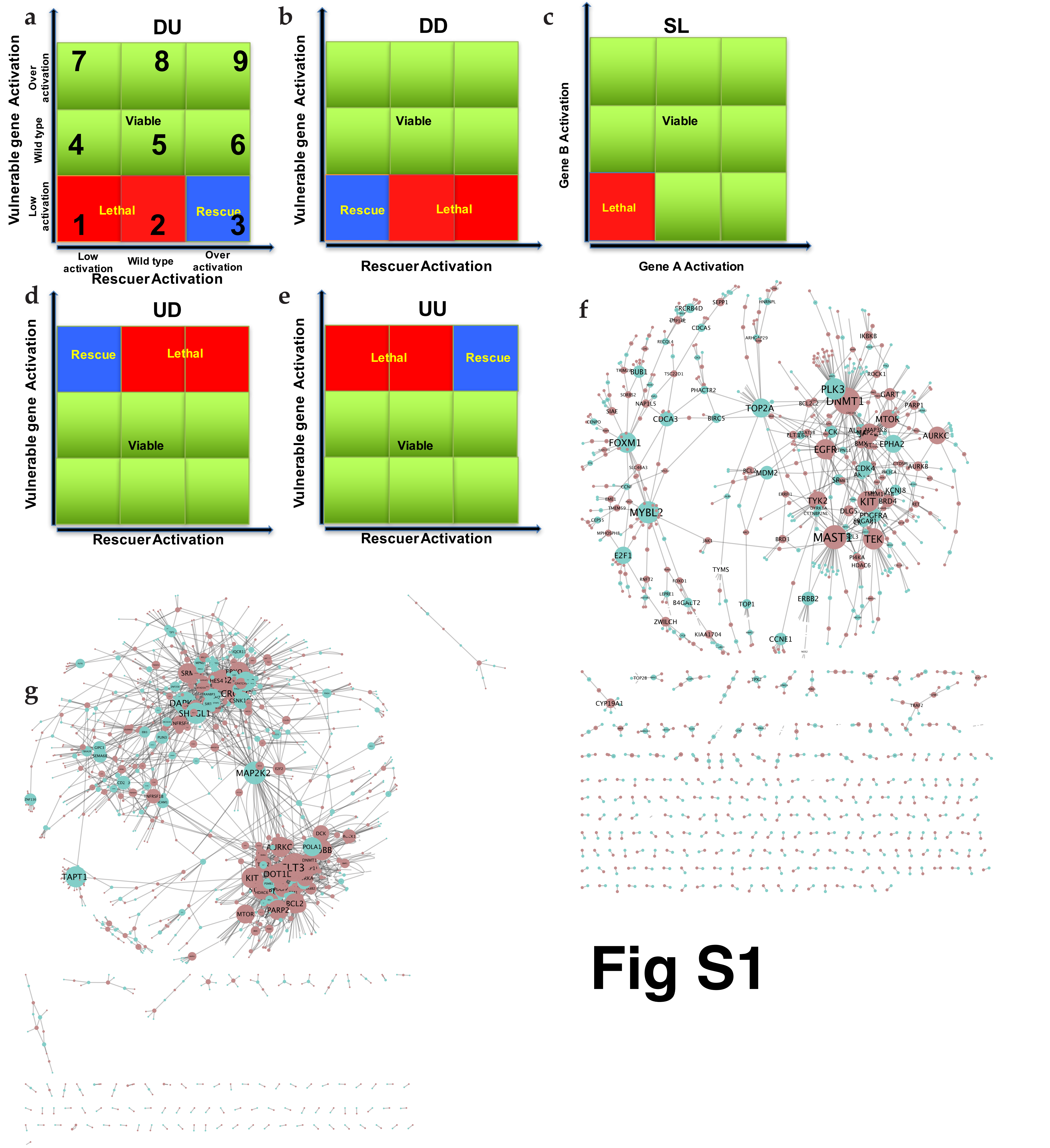
a
DU
Vulnerable gene Activation
Over activation
Wild type
Low activation
7
8
9
Viable
4
5
6
Lethal
Rescue
1
2
3
Low activation
Wild type
Over activation
Rescuer Activation
b
DD
Vulnerable gene Activation
Viable
Rescue
Lethal
Rescuer Activation
c
SL
Gene B Activation
Viable
Lethal
Gene A Activation
d
UD
Vulnerable gene Activation
Rescue
Lethal
Viable
Rescuer Activation
e
UU
Vulnerable gene Activation
Lethal
Rescue
Viable
Rescuer Activation
f
PLK3
DNMT1
TOP2A
FOXM1
CDCA3
MYBL2
E2F1
EGFR
MDM2
TYK2
KIT
MAST1
TEK
AURKC
EPHA2
MTOR
CDK4
ERBB2
TOP1
CCNE1
g
MAP2K2
TAPT1
AURKC
POLA1
KIT
MTOR
BCL2
PARP2

Fig S1

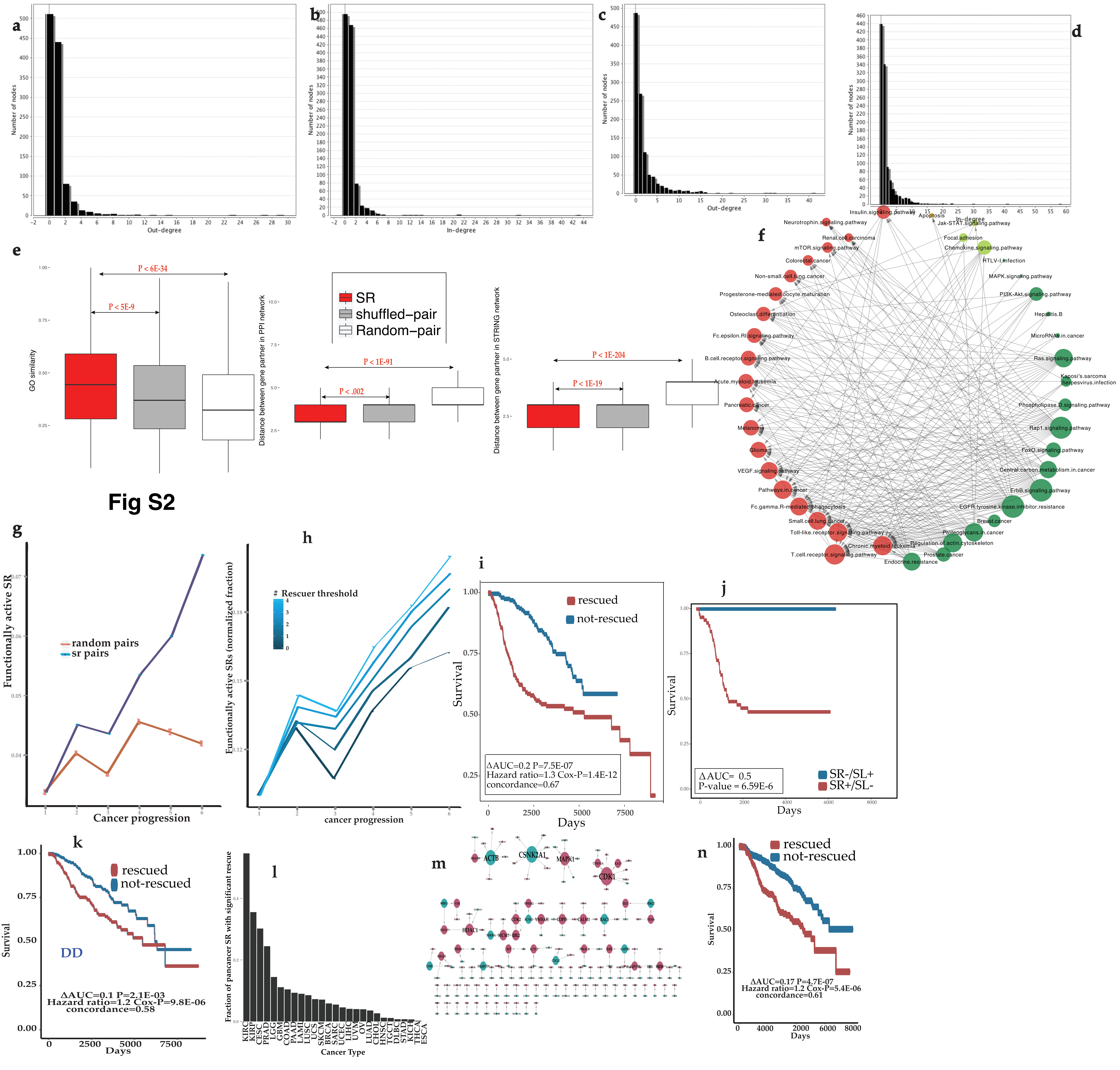
a
Number of nodes
Out-degree
b
Number of nodes
In-degree
c
Number of nodes
Out-degree
d
Number of nodes
In-degree
e
GO similarity
P < 6E-34
P < 5E-9
Distance between gene partner in PPI network
P < 1E-91
P < .002
SR
shuffled−pair
Random−pair
Distance between gene partner in STRING network
P < 1E-204
P < 1E-19
f
Insulin.signaling.pathway
Apoptosis
Jak-STAT.signaling.pathway
Neurotrophin.signaling.pathway
Renal.cell.carcinoma
Focal.adhesion
mTOR.signaling.pathway
Chemokine.signaling.pathway
Colorectal.cancer
HTLV-I.infection
Non-small.cell.lung.cancer
MAPK.signaling.pathway
Progesterone-mediated.oocyte.maturation
PI3K-Akt.signaling.pathway
Osteoclast.differentiation
Hepatitis.B
Fc.epsilon.RI.signaling.pathway
MicroRNAs.in.cancer
B.cell.receptor.signaling.pathway
Ras.signaling.pathway
Acute.myeloid.leukemia
Kaposi's.sarcoma
herpesvirus.infection
Pancreatic.cancer
Phospholipase.D.signaling.pathway
Melanoma
Rap1.signaling.pathway
Glioma
FoxO.signaling.pathway
VEGF.signaling.pathway
Central.carbon.metabolism.in.cancer
Pathways.in.cancer
ErbB.signaling.pathway
Fc.gamma.R-mediated.phagocytosis
EGFR.tyrosine.kinase.inhibitor.resistance
Small.cell.lung.cancer
Breast.cancer
Toll-like.receptor.signaling.pathway
Proteoglycans.in.cancer
Chronic.myeloid.leukemia
Regulation.of.actin.cytoskeleton
T.cell.receptor.signaling.pathway
Prostate.cancer
Endocrine.resistance
g
Functionally active SR
random pairs
sr pairs
Cancer progression
h
Functionally active SRs (normalized fraction)
Rescuer threshold
cancer progression
i
rescued
not-rescued
Survival
ΔAUC=0.2 P=7.5E-07
Hazard ratio=1.3 Cox-P=1.4E-12
concordance=0.67
Days
j
Survival
ΔAUC= 0.5
P-value = 6.59E-6
SR-/SL+
SR+/SL-
Days
k
rescued
not-rescued
DD
Survival
ΔAUC=0.1 P=2.1E-03
Hazard ratio=1.2 Cox-P=9.8E-06
concordance=0.58
Days
l
Fraction of pancancer SR with significant rescue
KIRC
KIRP
CESC
PRAD
LGG
GBM
COAD
PAAD
LAML
LUSC
UCS
SKCM
BRCA
SARC
UCEC
LIHC
UVM
OV
LUAD
CHOL
HNSC
TGCT
DLBC
STAD
KICH
THCA
ESCA
Cancer Type
m
ACTB
CSNK2A1
MAPK1
CDK1
HDAC1
n
rescued
not-rescued
Survival
ΔAUC=0.17 P=4.7E-07
Hazard ratio=1.2 Cox-P=5.4E-06
concordance=0.61
Days

Fig S2

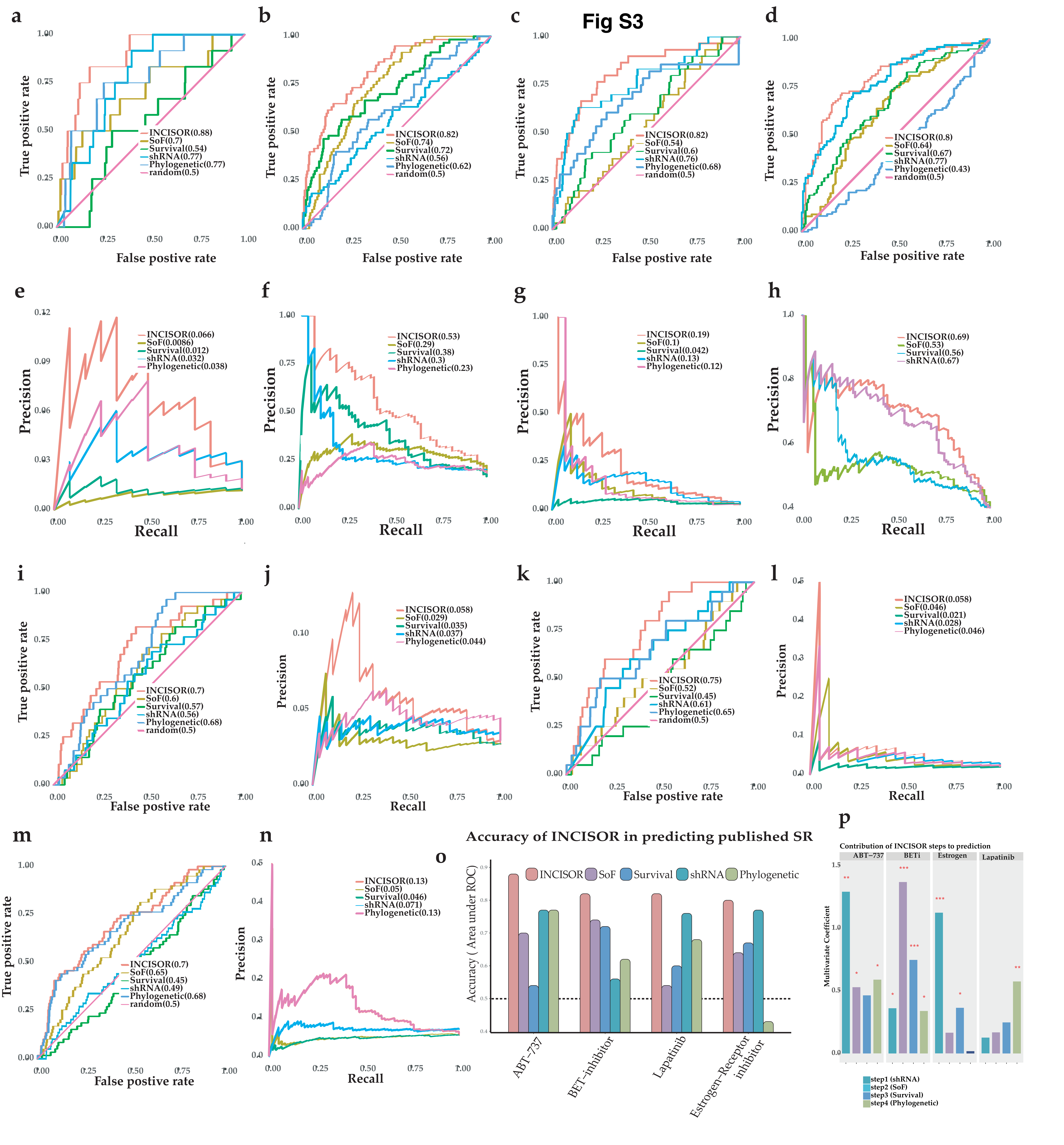

Fig S3
a
b
c
d
True positive rate
False postive rate
INCISOR(0.88)
SoF(0.7)
Survival(0.54)
shRNA(0.77)
Phylogenetic(0.77)
random(0.5)
INCISOR(0.82)
SoF(0.74)
Survival(0.72)
shRNA(0.56)
Phylogenetic(0.62)
random(0.5)
INCISOR(0.82)
SoF(0.54)
Survival(0.6)
shRNA(0.76)
Phylogenetic(0.68)
random(0.5)
INCISOR(0.8)
SoF(0.64)
Survival(0.67)
shRNA(0.77)
Phylogenetic(0.43)
random(0.5)
e
f
g
h
Precision
Recall
INCISOR(0.066)
SoF(0.0086)
Survival(0.012)
shRNA(0.032)
Phylogenetic(0.038)
INCISOR(0.53)
SoF(0.29)
Survival(0.38)
shRNA(0.3)
Phylogenetic(0.23)
INCISOR(0.19)
SoF(0.1)
Survival(0.042)
shRNA(0.13)
Phylogenetic(0.12)
INCISOR(0.69)
SoF(0.53)
Survival(0.56)
shRNA(0.67)
i
j
k
l
INCISOR(0.7)
SoF(0.6)
Survival(0.57)
shRNA(0.56)
Phylogenetic(0.68)
random(0.5)
INCISOR(0.058)
SoF(0.029)
Survival(0.035)
shRNA(0.037)
Phylogenetic(0.044)
INCISOR(0.75)
SoF(0.52)
Survival(0.45)
shRNA(0.61)
Phylogenetic(0.65)
random(0.5)
INCISOR(0.058)
SoF(0.046)
Survival(0.021)
shRNA(0.028)
Phylogenetic(0.046)
m
n
INCISOR(0.7)
SoF(0.65)
Survival(0.45)
shRNA(0.49)
Phylogenetic(0.68)
random(0.5)
INCISOR(0.13)
SoF(0.05)
Survival(0.046)
shRNA(0.071)
Phylogenetic(0.13)
o
Accuracy of INCISOR in predicting published SR
INCISOR
SoF
Survival
shRNA
Phylogenetic
Accuracy (Area under ROC)
ABT−737
BET−inhibitor
Lapatinib
Estrogen−Receptor inhibitor
p
Contribution of INCISOR steps to prediction
ABT−737
BETi
Estrogen
Lapatinib
Multivariate Coefficient
step1 (shRNA)
step2 (SoF)
step3 (Survival)
step4 (Phylogenetic)

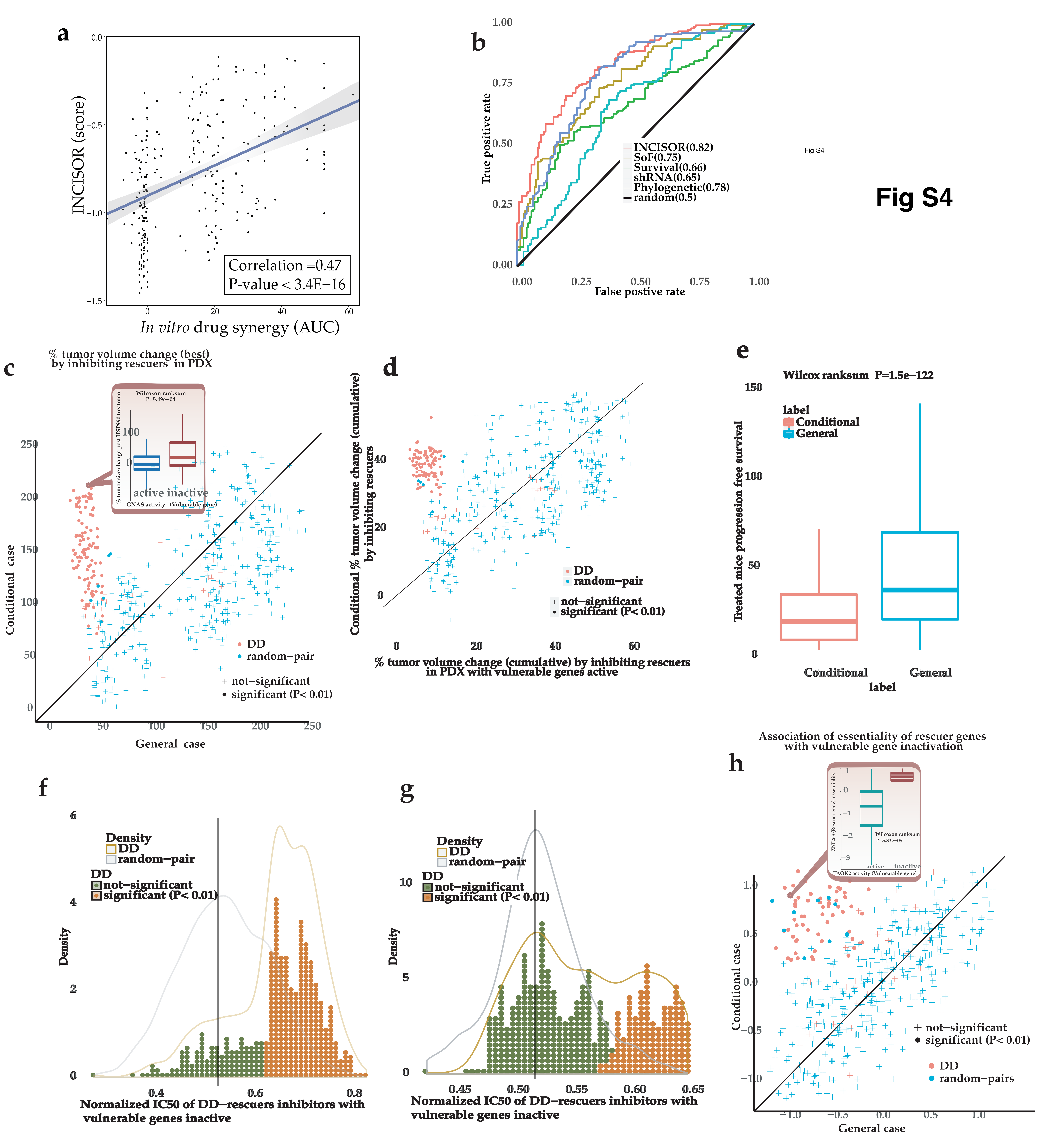
a
INCISOR (score)
In vitro drug synergy (AUC)
Correlation =0.47
P-value < 3.4E−16
b
True positive rate
False postive rate
INCISOR(0.82)
SoF(0.75)
Survival(0.66)
shRNA(0.65)
Phylogenetic(0.78)
random(0.5)
Fig S4
c
% tumor volume change (best)
by inhibiting rescuers in PDX
Wilcoxon ranksum
P=5.49e−04
% tumor size change post HSP990 treatment
active inactive
GNAS activity (Vulnerable gene)
Conditional case
General case
DD
random−pair
not−significant
significant (P< 0.01)
d
Conditional % tumor volume change (cumulative)
by inhibiting rescuers
% tumor volume change (cumulative) by inhibiting rescuers
in PDX with vulnerable genes active
DD
random−pair
not−significant
significant (P< 0.01)
e
Wilcox ranksum P=1.5e−122
label
Conditional
General
Treated mice progression free survival
Conditional
General
label
f
Density
DD
random−pair
DD
not−significant
significant (P< 0.01)
Density
Normalized IC50 of DD−rescuers inhibitors with
vulnerable genes inactive
g
Density
DD
random−pair
DD
not−significant
significant (P< 0.01)
Density
Normalized IC50 of DD−rescuers inhibitors with
vulnerable genes inactive
h
Association of essentiality of rescuer genes
with vulnerable gene inactivation
ZNF263 (Rescuer gene) essentiality
Wilcoxon ranksum
P=5.83e−05
active inactive
TAOK2 activity (Vulnerable gene)
Conditional case
General case
not−significant
significant (P< 0.01)
DD
random−pairs

Fig S4

Fig S5

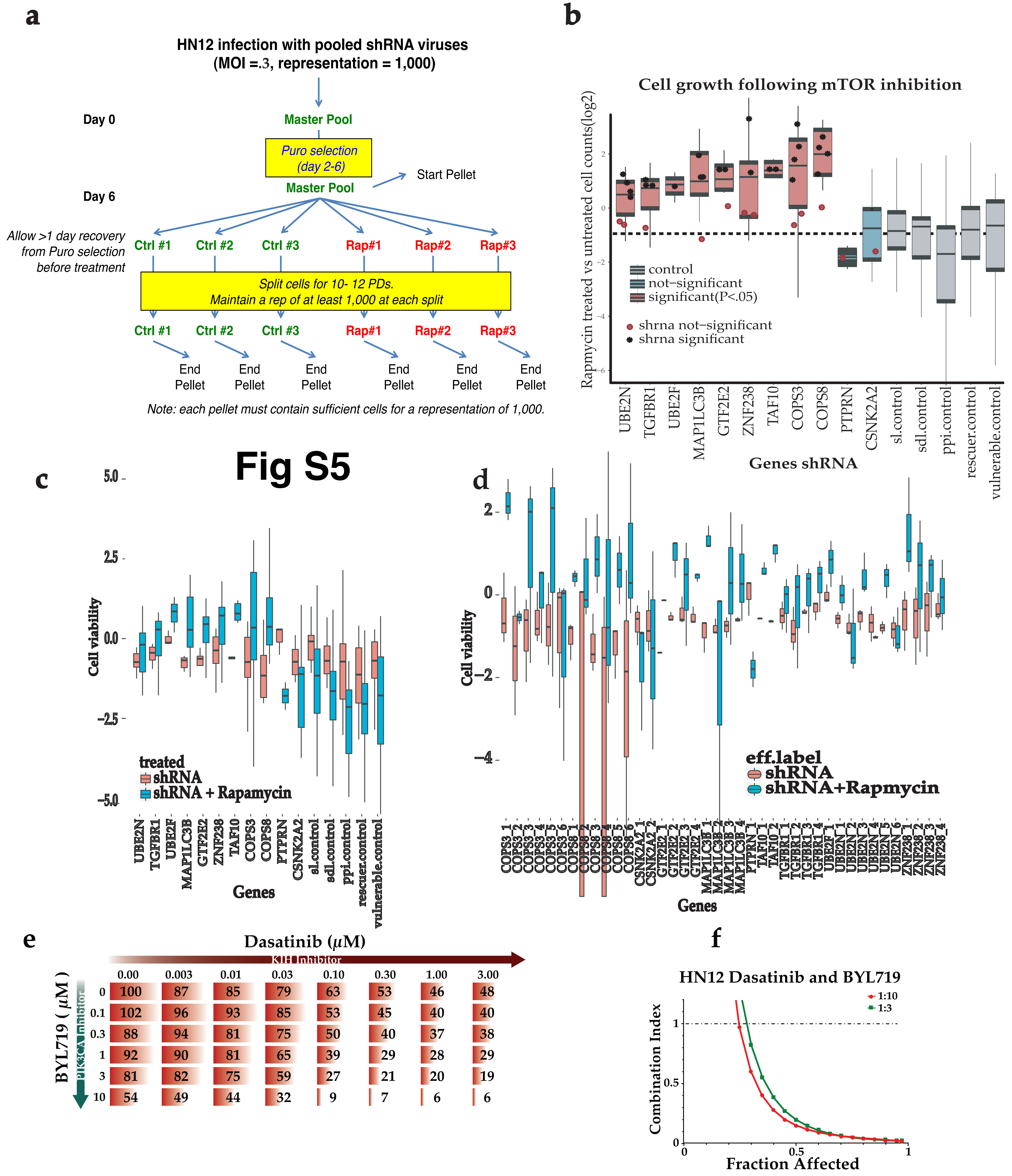
a
HN12 infection with pooled shRNA viruses
(MOI =.3, representation = 1,000)
Day 0
Master Pool
Puro selection
(day 2-6)
Day 6
Master Pool
Start Pellet
Allow >1 day recovery from Puro selection before treatment
Ctrl #1
Ctrl #2
Ctrl #3
Rap#1
Rap#2
Rap#3
Split cells for 10- 12 PDs.
Maintain a rep of at least 1,000 at each split
End Pellet
Note: each pellet must contain sufficient cells for a representation of 1,000.
b
Cell growth following mTOR inhibition
Rapmycin treated vs untreated cell counts(log2)
control
not−significant
significant(P<.05)
shrna not−significant
shrna significant
UBE2N
TGFBR1
UBE2F
MAP1LC3B
GTF2E2
ZNF238
TAF10
COPS3
COPS8
PTPRN
CSNK2A2
sl.control
sdl.control
ppi.control
rescuer.control
vulnerable.control
Genes shRNA
c
Cell viability
treated
shRNA
shRNA + Rapamycin
Genes
d
Cell viability
eff.label
shRNA
shRNA+Rapmycin
Genes
e
Dasatinib (μM)
KIH Inhibitor
BYL719 (μM)
PIK3CA Inhibitor
0.00 0.003 0.01 0.03 0.10 0.30 1.00 3.00
0: 100 87 85 79 63 53 46 48
0.1: 102 96 93 85 53 45 40 40
0.3: 88 94 81 75 50 40 37 38
1: 92 90 81 65 39 29 28 29
3: 81 82 75 59 27 21 20 19
10: 54 49 44 32 9 7 6 6
f
HN12 Dasatinib and BYL719
1:10
1:3
Combination Index
Fraction Affected

a

Dasatinib and MK2206

Cal27	0.00	0.003	0.01	0.03	0.10	0.30	1.00	3.00	Dasatinib(μM)
0	100	92	89	86	74	60	49	42	
0.1	92	86	86	85	66	51	43	28	
0.3	89	87	89	83	62	49	37	25	
1	80	79	77	75	51	39	29	20	
3	71	63	59	56	37	29	22	16	
10	45	43	42	36	20	14	9	5	
MK2206(μM)									

HN12	0.00	0.003	0.01	0.03	0.10	0.30	1.00	3.00	Dasatinib(μM)
0	100	98	93	88	68	58	56	51	
0.1	101	96	89	77	67	57	53	47	
0.3	94	93	85	73	57	46	46	40	
1	88	87	74	68	47	42	39	35	
3	85	79	70	54	41	32	29	29	
10	60	59	48	35	22	16	12	12	
MK2206(μM)									

Cal33	0.00	0.003	0.01	0.03	0.10	0.30	1.00	3.00	Dasatinib(μM)
0	100	91	81	74	59	59	52	51	
0.1	96	80	74	57	44	34	32	35	
0.3	69	66	51	46	32	24	24	25	
1	54	52	43	33	24	19	17	17	
3	38	38	30	23	17	14	14	13	
10	20	19	17	13	10	9	10	8	
MK2206(μM)									

SCC47	0.00	0.003	0.01	0.03	0.10	0.30	1.00	3.00	Dasatinib(μM)
0	100	100	95	86	50	38	27	22	
0.1	100	97	96	86	55	37	24	19	
0.3	91	93	89	74	49	32	21	18	
1	88	85	80	70	45	30	21	18	
3	79	76	69	62	36	25	18	15	
10	46	35	35	24	17	15	11	11	
MK2206(μM)									

Detroit 562	0.00	0.003	0.01	0.03	0.10	0.30	1.00	3.00	Dasatinib(μM)
0	100	89	82	61	31	26	26	23	
0.1	105	89	74	53	23	18	15	14	
0.3	86	74	62	36	15	12	10	7	
1	67	57	44	25	10	8	6	4	
3	48	37	31	18	8	7	6	4	
10	25	21	17	11	5	4	2	1	
MK2206(μM)									

Dasatinib: 50-140 mg once daily
Reference: Rapamycin 5 mg once daily
MK2206: 40-200 mg once daily

b

Dasatinib and BYL719

Cal27	0	0.003	0.01	0.03	0.1	0.3	1	3	Dasatinib(μM)
0	100	101	95	95	82	62	57	49	
0.1	97	101	96	99	78	58	51	44	
0.3	103	104	102	94	75	50	44	33	
1	97	101	94	86	60	40	31	24	
3	83	79	79	76	52	33	23	16	
10	54	57	50	41	23	13	10	7	
BYL719(μM)									

HN12	0.00	0.003	0.01	0.03	0.10	0.30	1.00	3.00	Dasatinib(μM)
0	100	87	85	79	63	53	46	48	
0.1	102	96	93	85	53	45	40	40	
0.3	88	94	81	75	50	40	37	38	
1	92	90	81	65	39	29	28	29	
3	81	82	75	59	27	21	20	19	
10	54	49	44	32	9	7	6	6	
BYL719(μM)									

Cal33	0.00	0.003	0.01	0.03	0.10	0.30	1.00	3.00	Dasatinib(μM)
0	100	98	94	77	68	66	65	54	
0.1	96	90	83	70	61	57	59	46	
0.3	85	84	75	65	49	46	46	36	
1	54	58	50	36	26	26	24	20	
3	26	26	23	20	14	12	15	11	
10	14	14	13	10	5	5	5	4	
BYL719(μM)									

SCC47	0.00	0.003	0.01	0.03	0.10	0.30	1.00	3.00	Dasatinib(μM)
0	100	91	92	80	49	36	27	21	
0.1	102	96	93	79	42	30	23	18	
0.3	99	90	87	72	32	22	19	15	
1	92	82	80	63	25	19	17	14	
3	81	71	72	51	20	17	15	13	
10	53	48	46	31	12	11	10	8	
BYL719(μM)									

Detroit 562	0.00	0.003	0.01	0.03	0.10	0.30	1.00	3.00	Dasatinib(μM)
0	100	86	73	60	32	25	23	22	
0.1	101	90	75	52	24	20	20	20	
0.3	83	80	58	36	17	15	15	13	
1	55	45	33	20	10	8	7	4	
3	28	25	17	8	4	3	3	2	
10	10	8	6	2	2	1	1	1	
BYL719(μM)									

Dasatinib: 50-140 mg once daily
BYL719: 200-350 mg once daily
Reference: Rapamycin 5 mg once daily

c

Erlotinib and MK2206

Cal27	0.00	0.01	0.03	0.10	0.30	1.00	3.00	10.00	Erlotinib (μM)
0	100	92	101	90	78	65	57	36	
0.1	93	92	91	81	62	42	32	22	
0.3	88	82	81	68	50	34	25	17	
1	75	71	71	56	44	31	23	15	
3	61	62	56	46	36	26	21	15	
10	44	44	41	32	27	19	15	11	
MK2206 (μM)									

HN12	0.00	0.01	0.03	0.10	0.30	1.00	3.00	10.00	Erlotinib (μM)
0	100	92	103	100	91	89	67	54	
0.1	101	110	104	101	102	86	64	44	
0.3	97	93	92	89	89	76	54	42	
1	96	95	91	91	88	66	53	40	
3	84	85	78	78	68	59	44	37	
10	57	56	51	46	42	36	32	27	
MK2206 (μM)									

Cal33	0.00	0.01	0.03	0.10	0.30	1.00	3.00	10.00	Erlotinib (μM)
0	100	99	98	90	88	80	60	47	
0.1	94	95	90	89	80	68	48	34	
0.3	76	74	65	65	60	46	34	29	
1	55	53	47	45	40	32	24	18	
3	39	38	32	30	31	24	18	13	
10	18	17	18	18	18	16	14	9	
MK2206 (μM)									

SCC47	0.00	0.01	0.03	0.10	0.30	1.00	3.00	10.00	Erlotinib (μM)
0	100	96	86	86	80	63	41	36	
0.1	92	88	86	81	71	53	38	30	
0.3	85	84	81	75	64	52	37	29	
1	80	81	74	69	60	51	37	29	
3	79	77	77	68	58	51	36	27	
10	46	48	48	45	39	36	30	22	
MK2206 (μM)									

Detroit 562	0.00	0.01	0.03	0.10	0.30	1.00	3.00	10.00	Erlotinib (μM)
0	100	91	91	85	74	63	41	28	
0.1	103	99	90	81	68	47	30	20	
0.3	75	78	66	58	45	33	21	13	
1	60	59	55	49	39	30	18	9	
3	43	44	39	34	29	23	14	7	
10	18	18	16	15	13	11	7	3	
MK2206 (μM)									

Erlotinib: 150-300 mg once daily
MK2206: 40-200 mg once daily
Reference: Rapamycin 5 mg once daily

d

BYL719 and Rapamycin

Cal27	0.00	0.01	0.03	0.10	0.30	1.00	3.00	10.00	BYL719(μM)
0	100	90	86	88	83	76	63	43	
1	68	60	57	54	51	46	32	24	
3	65	59	57	52	51	40	27	24	
10	66	62	61	53	54	41	30	21	
30	67	62	58	53	51	39	30	22	
100	62	59	52	56	51	40	33	23	
300	38	38	35	36	31	26	19	13	
Rapamycin (nM)									

HN12	0.00	0.01	0.03	0.10	0.30	1.00	3.00	10.00	BYL719(μM)
0	100	96	95	91	85	85	73	50	
1	77	75	76	71	69	62	54	37	
3	75	71	72	68	65	56	48	32	
10	70	72	69	66	67	57	48	35	
30	74	66	73	72	69	54	49	32	
100	72	70	71	67	65	51	44	30	
300	47	47	46	42	41	35	33	23	
Rapamycin (nM)									

Cal33	0.00	0.01	0.03	0.10	0.30	1.00	3.00	10.00	BYL719(μM)
0	100	96	92	78	75	56	25	14	
1	62	54	56	57	45	34	20	14	
3	58	55	56	54	43	30	18	13	
10	50	53	44	46	43	30	20	12	
30	47	45	42	42	40	28	19	10	
100	45	43	42	42	39	24	16	11	
300	37	38	38	33	28	19	12	9	
Rapamycin (nM)									

SCC47	0.00	0.01	0.03	0.10	0.30	1.00	3.00	10.00	BYL719(μM)
0	100	93	94	94	81	76	67	46	
1	85	82	84	80	77	65	51	31	
3	86	80	84	80	76	67	52	32	
10	82	83	82	79	76	67	52	33	
30	87	85	83	83	77	68	54	35	
100	81	79	78	77	73	67	49	34	
300	56	55	53	51	46	41	31	21	
Rapamycin (nM)									

Detroit 562	0.00	0.01	0.03	0.10	0.30	1.00	3.00	10.00	BYL719(μM)
0	100	95	92	84	73	48	23	8	
1	90	87	86	77	68	38	21	9	
3	89	91	82	79	63	38	21	10	
10	82	81	76	75	66	41	23	9	
30	81	76	79	73	55	39	20	9	
100	66	65	67	66	54	39	20	9	
300	42	42	41	42	32	21	10	5	
Rapamycin (nM)									

BYL719: 200-350 mg once daily
Rapamycin 5 mg once daily

e

BYL719 and ink128

Cal27	0.00	0.01	0.03	0.10	0.30	1.00	3.00	10.00	BYL719(μM)
0	100	94	78	81	81	66	67	49	
1	94	94	76	80	70	62	55	43	
3	83	84	66	71	62	50	40	30	
10	55	51	43	41	36	29	25	22	
30	32	31	25	25	21	18	16	18	
100	26	25	20	21	19	17	14	15	
300	19	17	14	15	14	13	10	10	
ink128 (nM)									

HN12	0.00	0.01	0.03	0.10	0.30	1.00	3.00	10.00	BYL719(μM)
0	100	96	96	94	86	82	75	53	
1	99	96	93	91	87	79	68	51	
3	89	85	84	81	77	66	56	41	
10	65	66	62	60	57	47	42	33	
30	48	51	48	46	42	34	31	25	
100	40	40	38	36	33	30	28	24	
300	24	24	24	23	22	20	19	16	
ink128 (nM)									

Cal33	0.00	0.01	0.03	0.10	0.30	1.00	3.00	10.00	BYL719(μM)
0	100	88	96	94	85	58	25	14	
1	94	89	84	80	80	45	25	15	
3	76	78	71	64	57	32	22	14	
10	51	49	45	42	35	24	19	13	
30	33	33	26	31	29	22	18	12	
100	24	25	24	24	24	19	16	10	
300	17	15	16	14	12	11	9	8	
ink128 (nM)									

SCC47	0.00	0.01	0.03	0.10	0.30	1.00	3.00	10.00	BYL719(μM)
0	100	99	95	93	87	82	66	53	
1	109	104	105	105	97	86	76	57	
3	103	101	100	97	88	80	66	51	
10	87	84	79	72	66	56	47	39	
30	60	55	54	50	45	39	35	32	
100	41	41	39	37	35	30	27	27	
300	19	19	18	19	18	15	15	15	
ink128 (nM)									

Detroit 562	0.00	0.01	0.03	0.10	0.30	1.00	3.00	10.00	BYL719(μM)
0	100	94	94	92	77	47	23	9	
1	101	101	96	96	76	51	26	11	
3	95	93	90	83	64	38	19	10	
10	64	64	62	54	41	25	18	9	
30	41	40	39	34	29	20	14	8	
100	31	30	30	27	23	18	12	8	
300	15	14	15	14	11	8	7	4	
ink128 (nM)									

BYL719: 200-350 mg once daily
ink128: 15-30 mg once daily
Reference: Rapamycin 5 mg once daily

f

PND1186 and Rapamycin

Cal27	0	0.01	0.03	0.10	0.30	1.00	3.00	10.00	PND1186 (μM)
0	100	100	104	100	99	95	82	58	
1	68	66	65	64	61	60	55	56	
3	68	67	73	68	63	63	56	56	
10	67	67	69	65	61	62	54	53	
30	69	68	66	65	63	59	53	50	
100	68	62	64	63	55	58	52	56	
300	46	43	42	41	39	39	36	36	
Rapa (nM)									

HN12	0	0.01	0.03	0.1	0.3	1	3	10	PND1186 (μM)
0	100	103	100	95	98	79	74	68	
1	80	75	74	71	74	59	53	49	
3	78	80	68	71	74	68	57	53	
10	77	79	63	71	71	68	56	48	
30	73	74	68	76	70	69	59	49	
100	78	73	70	70	69	65	62	47	
300	52	50	48	49	49	53	41	38	
Rapa (nM)									

Cal33	0	0.01	0.03	0.1	0.3	1	3	10	PND1186 (μM)
0	100	88	69	76	68	61	60	50	
1	61	52	48	49	46	45	44	36	
3	57	52	49	49	42	44	38	38	
10	54	49	41	46	43	46	46	38	
30	52	56	47	54	44	50	41	34	
100	47	55	43	50	44	45	40	34	
300	38	34	34	34	31	30	30	31	
Rapa (nM)									

SCC47	0.00	0.01	0.03	0.10	0.30	1.00	3.00	10.00	BYL719(μM)
0	100	99	95	98	89	78	66	72	
1	83	86	84	84	76	70	62	62	
3	85	83	82	83	75	70	60	64	
10	81	84	81	81	73	70	64	60	
30	82	82	82	82	76	73	65	62	
100	77	78	79	79	72	67	63	58	
300	55	53	52	52	46	44	39	35	
Rapamycin (nM)									

Detroit 562	0	0.01	0.03	0.1	0.3	1	3	10	PND1186 (μM)
0	100	91	81	78	78	61	39	29	
1	78	81	69	68	69	56	34	31	
3	81	74	65	64	63	56	38	29	
10	76	76	66	62	60	54	39	28	
30	75	69	67	63	62	54	37	28	
100	68	70	61	63	60	52	37	26	
300	41	41	38	39	34	32	22	21	
Rapa (nM)									

g

PND1186 and ink128

Cal27	0	0.01	0.03	0.10	0.30	1.00	3.00	10.00	PND1186 (μM)
0	100	94	93	96	92	91	80	64	
1	94	90	79	86	84	82	71	57	
3	83	81	76	76	75	78	62	59	
10	55	51	48	50	47	46	40	51	
30	34	33	28	30	28	28	25	30	
100	28	25	24	24	22	22	19	22	
300	19	17	16	16	15	14	13	15	
ink128 (nM)									

HN12	0	0.01	0.03	0.10	0.30	1.00	3.00	10.00	PND1186 (μM)
0	100	98	89	90	89	86	72	62	
1	93	97	88	91	84	73	74	63	
3	91	89	88	83	83	69	64	57	
10	71	71	70	69	66	63	56	52	
30	51	52	49	49	48	46	41	39	
100	42	42	41	40	37	37	34	32	
300	28	28	26	27	24	22	20	22	
ink128 (nM)									

Cal33	0	0.01	0.03	0.10	0.30	1.00	3.00	10.00	PND1186 (μM)
0	100	93	84	86	84	66	68	56	
1	101	87	91	88	84	69	66	61	
3	86	86	81	81	74	70	64	55	
10	53	49	45	46	49	46	42	49	
30	39	36	35	36	37	36	36	38	
100	31	28	29	29	29	31	28	28	
300	19	17	17	17	16	16	16	18	
ink128 (nM)									

SCC47	0	0.01	0.03	0.10	0.30	1.00	3.00	10.00	PND1186 (μM)
0	100	95	88	96	95	86	75	70	
1	102	100	101	101	101	94	81	73	
3	100	96	97	98	96	92	77	71	
10	81	81	77	75	75	74	68	64	
30	58	54	51	53	50	50	52	54	
100	40	37	38	38	39	36	36	42	
300	21	20	20	20	20	20	20	24	
ink128 (nM)									

Detroit 562	0	0.01	0.03	0.10	0.30	1.00	3.00	10.00	PND1186 (μM)
0	100	100	92	99	99	75	43	41	
1	117	110	111	102	94	87	55	47	
3	95	90	89	90	96	74	48	44	
10	66	66	62	60	58	55	44	38	
30	47	44	40	39	41	37	33	29	
100	36	34	35	32	34	31	29	23	
300	28	26	27	26	24	23	21	20	
ink128 (nM)									

Dasatinib and MK2206

Fig S6

Fig S7

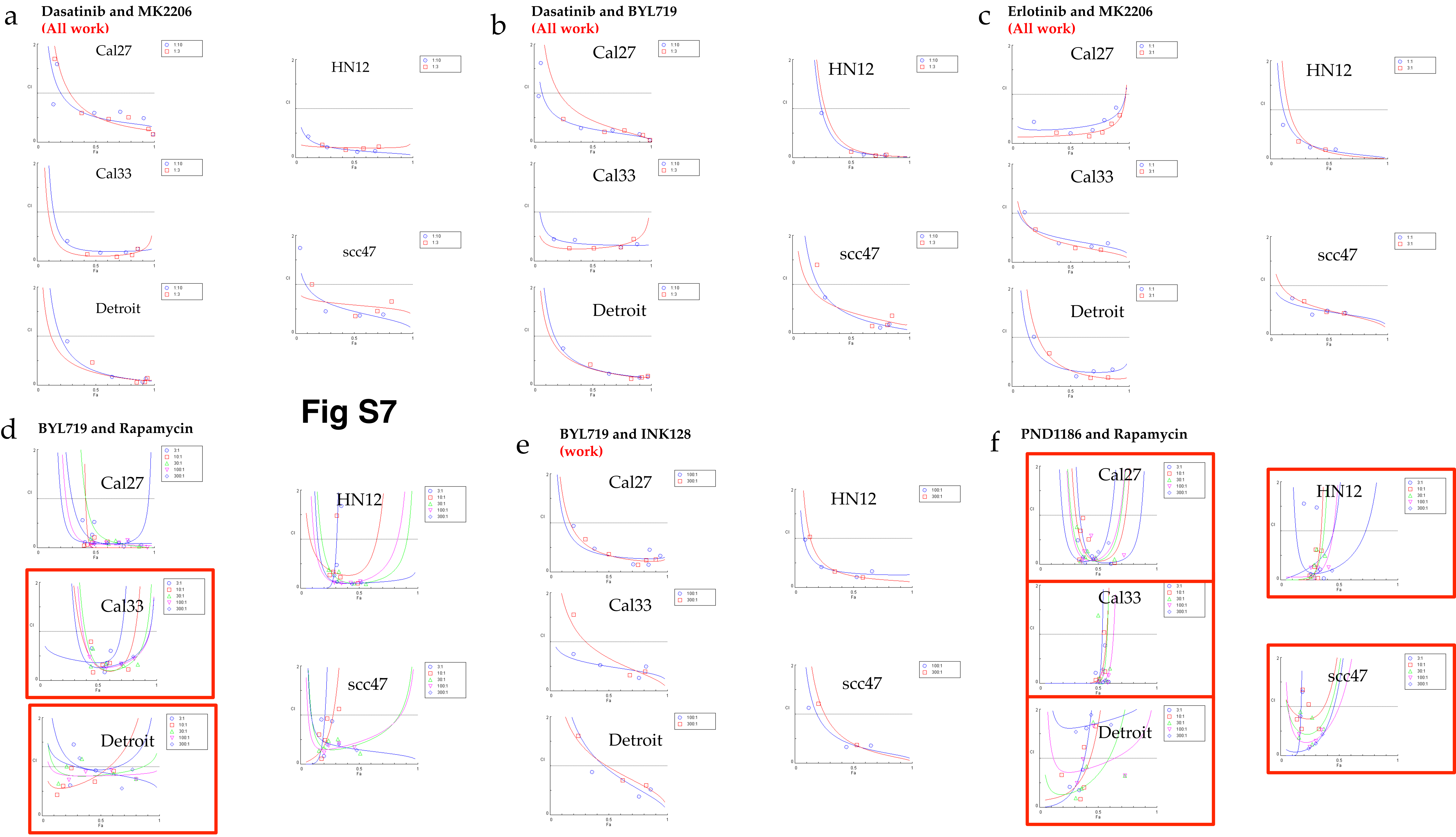

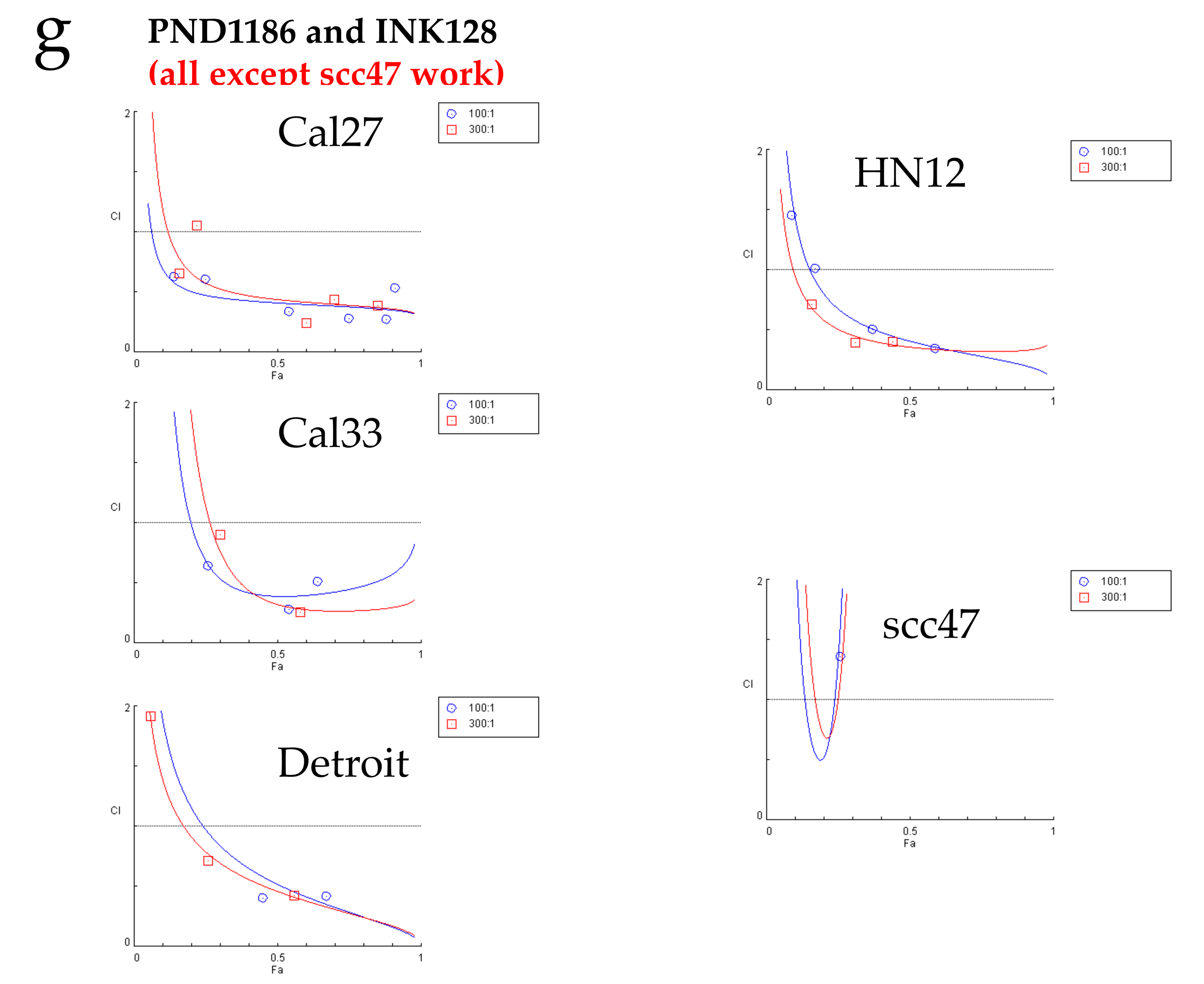

h

Rescuer gene	Vulnerable gene	Rescuer inhibitor (adjuvant)	Vulnerable gene inhibitor (Primary)	Cell lines (Synergism) Cal27	Cal33	Detroit 562	HN12	SCC47
AKT	KIT, SRC	MK2206	Dasatinib	0.51	0.25	0.28	0.17	0.5
PIK3CA	KIT, SRC	BYL719	Dasatinib	0.35	0.28	0.32	0.175	0.4
AKT	EGFR	MK2206	Erlotinib	0.18	0.4	0.35	0.16	0.48
mTOR	PIK3CA	Rapamycin	BYL719	0.128	0.3	0.866	0.167	0.38
mTOR	PIK3CA	ink128	BYL719	0.33	0.57	0.75	0.28	0.38
PTK2	mTOR	PND1186	Rapamycin	NAN	NAN	1.1	NAN	1.43
PTK2	mTOR	PND1186	ink128	0.42	0.35	0.47	0.37	NAN

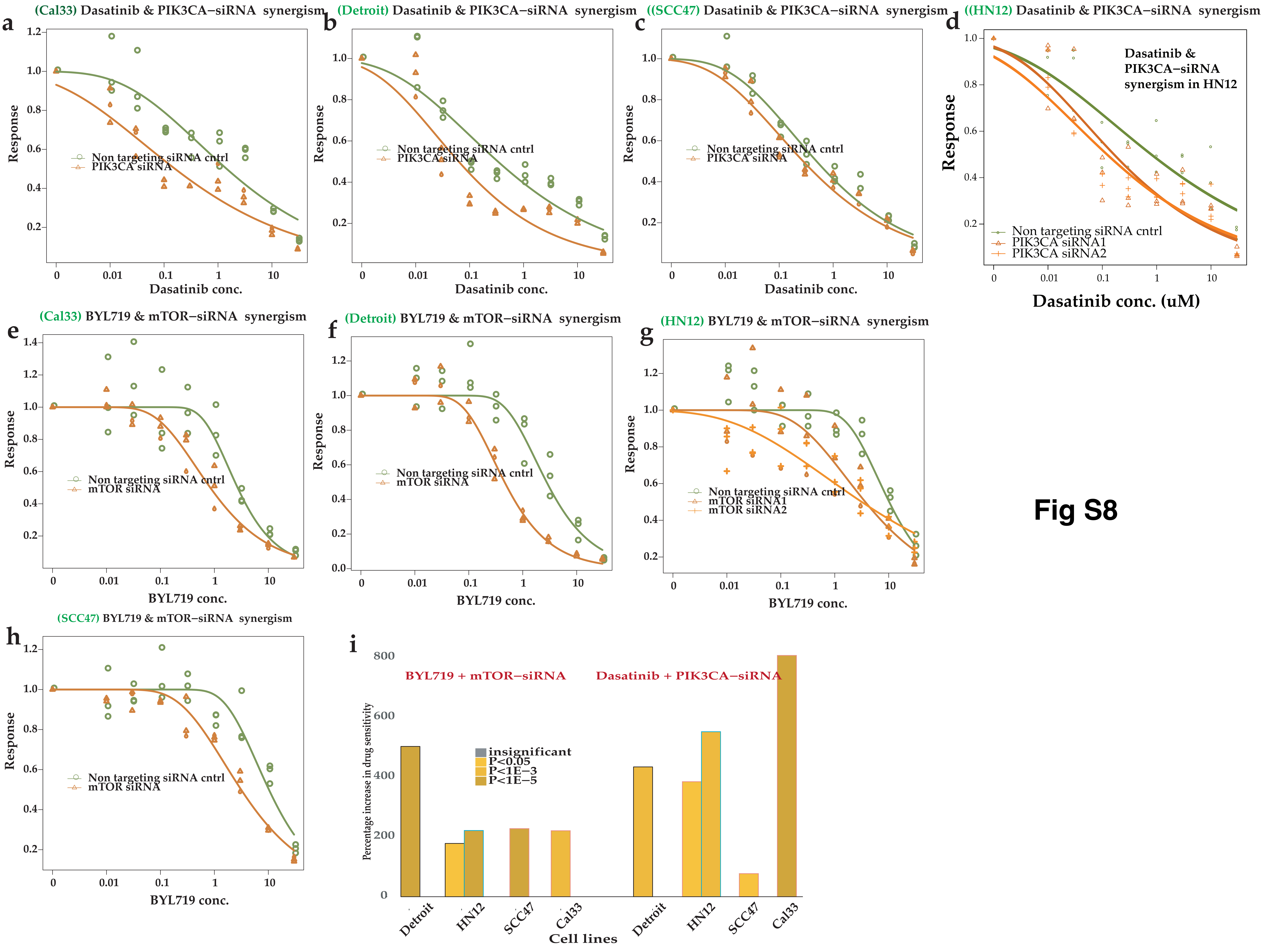
(Cal33) Dasatinib & PIK3CA−siRNA synergism
(Detroit) Dasatinib & PIK3CA−siRNA synergism
((SCC47) Dasatinib & PIK3CA−siRNA synergism
((HN12) Dasatinib & PIK3CA−siRNA synergism
Response
Dasatinib conc.
Non targeting siRNA cntrl
PIK3CA siRNA
Dasatinib & PIK3CA−siRNA synergism in HN12
PIK3CA siRNA1
PIK3CA siRNA2
Dasatinib conc. (uM)
(Cal33) BYL719 & mTOR−siRNA synergism
(Detroit) BYL719 & mTOR−siRNA synergism
(HN12) BYL719 & mTOR−siRNA synergism
(SCC47) BYL719 & mTOR−siRNA synergism
BYL719 conc.
mTOR siRNA
mTOR siRNA1
mTOR siRNA2
BYL719 + mTOR−siRNA
Dasatinib + PIK3CA−siRNA
Percentage increase in drug sensitivity
insignificant
P<0.05
P<1E−3
P<1E−5
Detroit
HN12
SCC47
Cal33
Cell lines

Fig S8

Fig S9

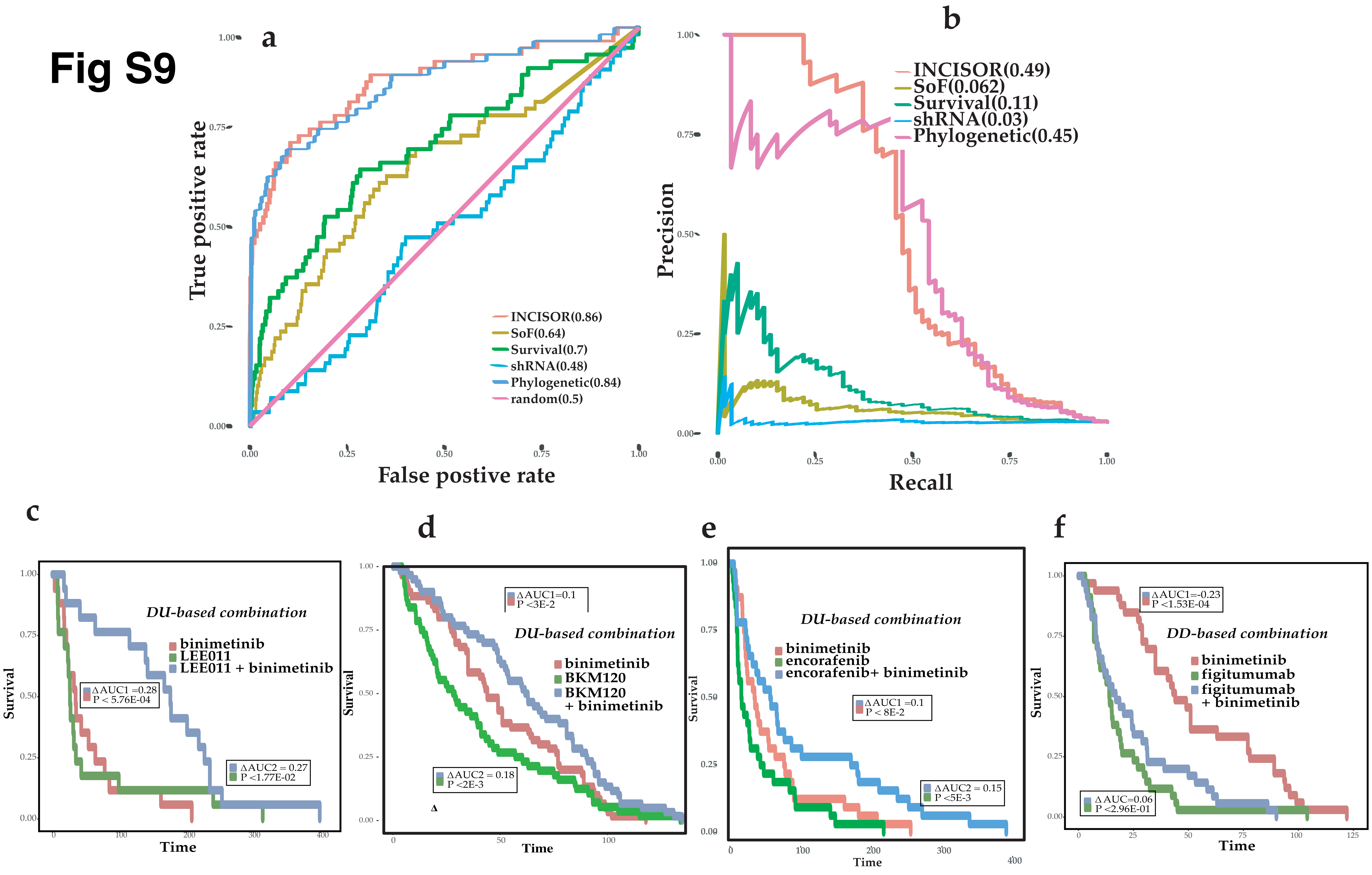
a
True positive rate
False postive rate
INCISOR(0.86)
SoF(0.64)
Survival(0.7)
shRNA(0.48)
Phylogenetic(0.84)
random(0.5)
b
Precision
Recall
INCISOR(0.49)
SoF(0.062)
Survival(0.11)
shRNA(0.03)
Phylogenetic(0.45)
c
DU-based combination
binimetinib
LEE011
LEE011 + binimetinib
ΔAUC1 =0.28
P < 5.76E-04
ΔAUC2 = 0.27
P <1.77E-02
Survival
Time
d
ΔAUC1=0.1
P <3E-2
DU-based combination
binimetinib
BKM120
BKM120
+ binimetinib
ΔAUC2 = 0.18
P <2E-3
Survival
Time
e
DU-based combination
binimetinib
encorafenib
encorafenib+ binimetinib
ΔAUC1 =0.1
P < 8E-2
ΔAUC2 = 0.15
P <5E-3
Survival
Time
f
ΔAUC1=-0.23
P <1.53E-04
DD-based combination
binimetinib
figitumumab
figitumumab
+ binimetinib
ΔAUC=0.06
P <2.96E-01
Survival
Time

Fig S10

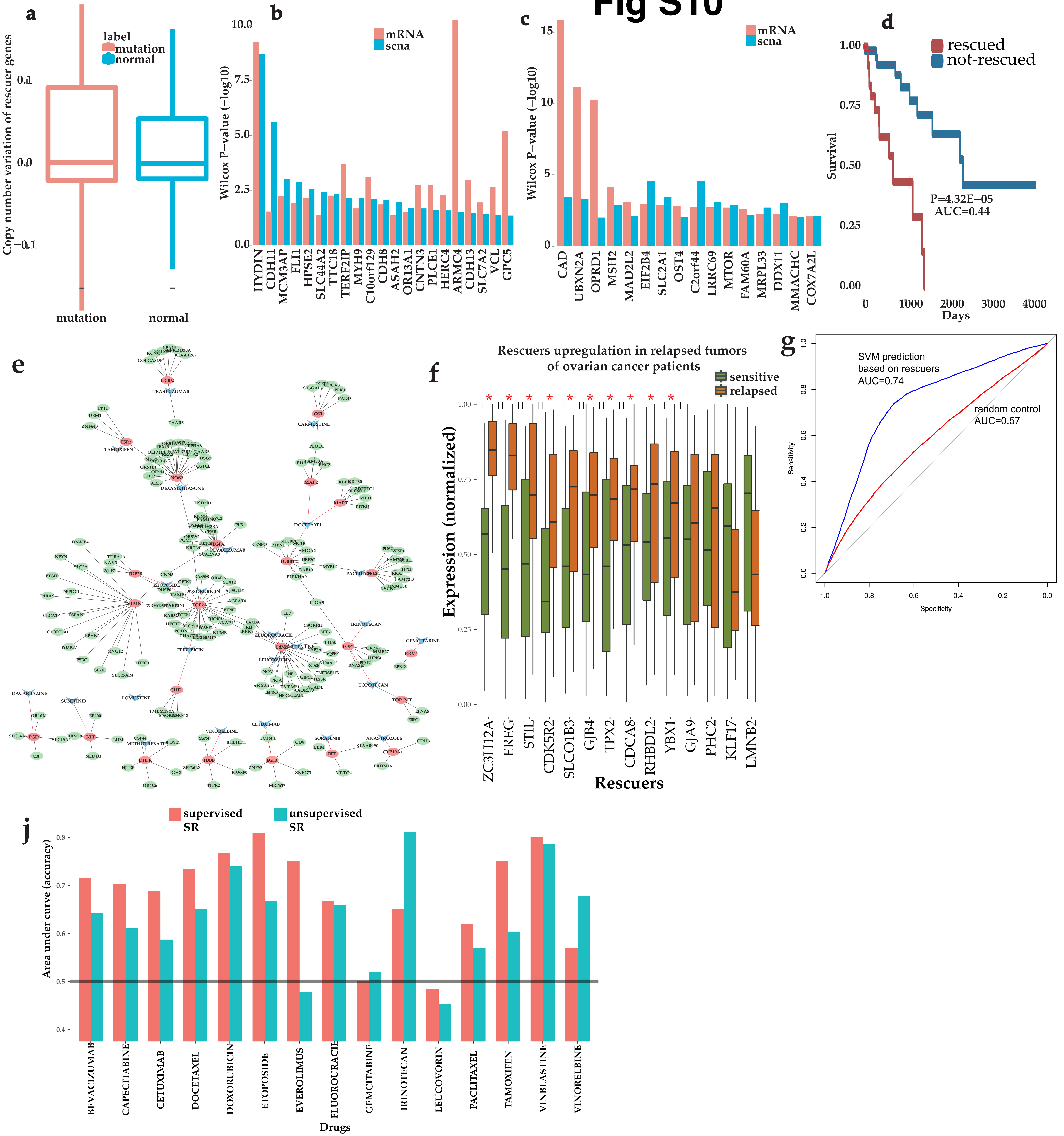
a
label
mutation
normal
Copy number variation of rescuer genes
0.1
0.0
−0.1
mutation
normal
b
mRNA
scna
Wilcox P−value (−log10)
10.0
7.5
5.0
2.5
0.0
HYDIN
CDH11
MCM3AP
FLI1
HPSE2
SLC44A2
TTC18
TERF2IP
MYH9
C10orf129
CDH8
ASAH2
OR13A1
CNTN3
PLCE1
HERC4
ARMC4
CDH13
SLC7A2
VCL
GPC5
c
mRNA
scna
Wilcox P−value (−log10)
15
10
5
0
CAD
UBXN2A
OPRD1
MSH2
MAD2L2
EIF2B4
SLC2A1
OST4
C2orf44
LRRC69
MTOR
FAM60A
MRPL33
DDX11
MMACHC
COX7A2L
d
rescued
not-rescued
Survival
1.00
0.75
0.50
0.25
0.00
P=4.32E−05
AUC=0.44
0
1000
2000
3000
4000
Days
e
f
Rescuers upregulation in relapsed tumors
of ovarian cancer patients
sensitive
relapsed
Expression (normalized)
1.00
0.75
0.50
0.25
0.00
ZC3H12A
EREG
STIL
CDK5R2
SLCO1B3
GJB4
TPX2
CDCA8
RHBDL2
YBX1
GJA9
PHC2
KLF17
LMNB2
Rescuers
g
SVM prediction
based on rescuers
AUC=0.74
random control
AUC=0.57
Sensitivity
Specificity
1.0
0.8
0.6
0.4
0.2
0.0
j
supervised
SR
unsupervised
SR
Area under curve (accuracy)
0.8
0.7
0.6
0.5
0.4
BEVACIZUMAB
CAPECITABINE
CETUXIMAB
DOCETAXEL
DOXORUBICIN
ETOPOSIDE
EVEROLIMUS
FLUOROURACIL
GEMCITABINE
IRINOTECAN
LEUCOVORIN
PACLITAXEL
TAMOXIFEN
VINBLASTINE
VINORELBINE
Drugs

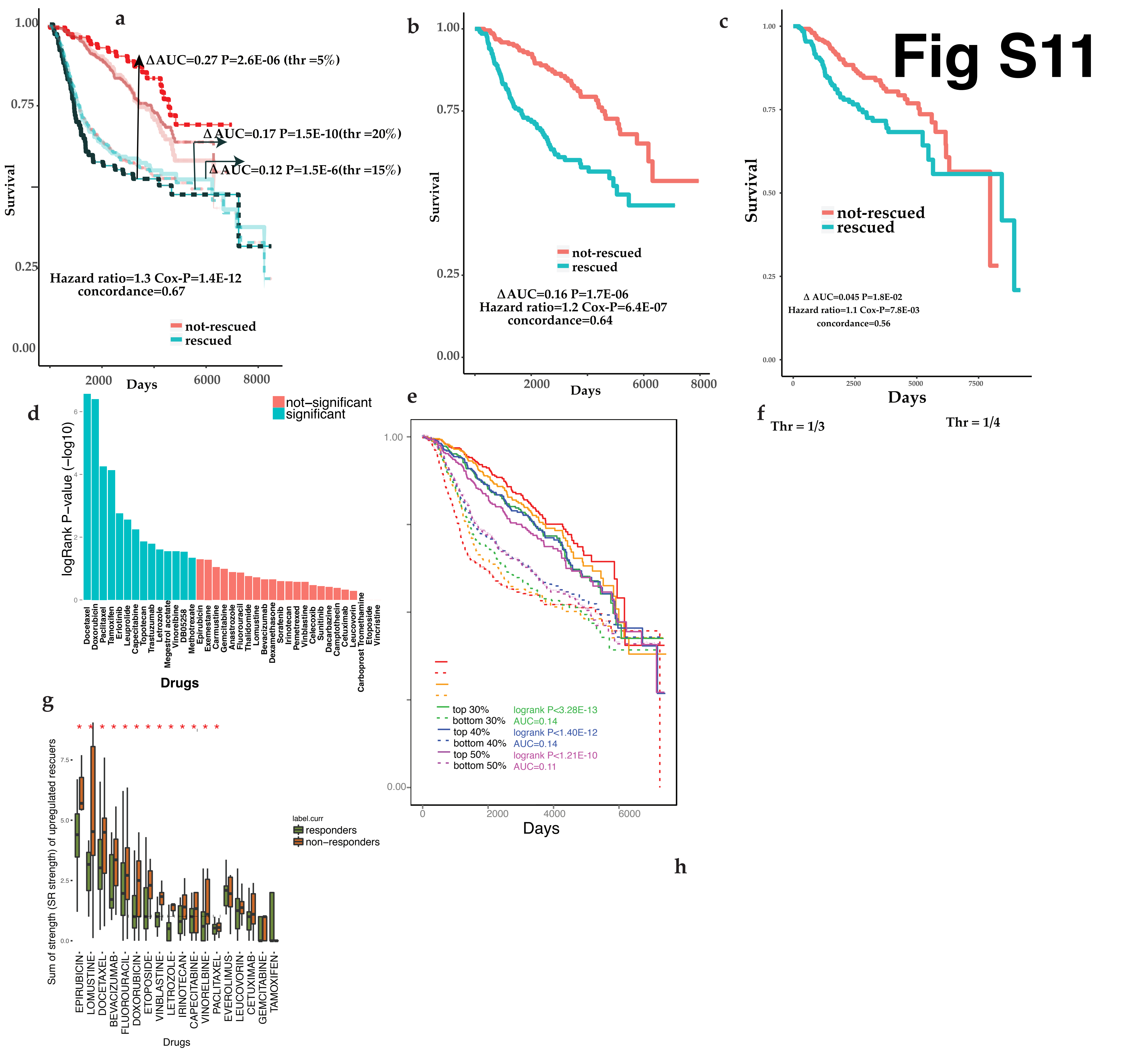
Fig S11
a
ΔAUC=0.27 P=2.6E-06 (thr =5%)
Δ AUC=0.17 P=1.5E-10(thr =20%)
Δ AUC=0.12 P=1.5E-6(thr =15%)
Hazard ratio=1.3 Cox-P=1.4E-12
concordance=0.67
not-rescued
rescued
Survival
Days
b
ΔAUC=0.16 P=1.7E-06
Hazard ratio=1.2 Cox-P=6.4E-07
concordance=0.64
not-rescued
rescued
c
Δ AUC=0.045 P=1.8E-02
Hazard ratio=1.1 Cox-P=7.8E-03
concordance=0.56
not-rescued
rescued
d
not−significant
significant
logRank P−value (−log10)
Drugs
Docetaxel Doxorubicin Paclitaxel Tamoxifen Erlotinib Leuprolide Capecitabine Topotecan Trastuzumab Letrozole Megestrol acetate Vinorelbine DB05258 Methotrexate Epirubicin Exemestane Carmustine Gemcitabine Anastrozole Fluorouracil Thalidomide Lomustine Bevacizumab Dexamethasone Sorafenib Irinotecan Pemetrexed Vinblastine Celecoxib Sunitinib Dacarbazine Camptothecin Cetuximab Leucovorin Carboprost Tromethamine Etoposide Vincristine
e
top 30% logrank P<3.28E-13
bottom 30% AUC=0.14
top 40% logrank P<1.40E-12
bottom 40% AUC=0.14
top 50% logrank P<1.21E-10
bottom 50% AUC=0.11
Days
f
Thr = 1/3
Thr = 1/4
g
Sum of strength (SR strength) of upregulated rescuers
label.curr
responders
non−responders
Drugs
EPIRUBICIN LOMUSTINE DOCETAXEL BEVACIZUMAB FLUOROURACIL DOXORUBICIN ETOPOSIDE VINBLASTINE LETROZOLE IRINOTECAN CAPECITABINE VINORELBINE PACLITAXEL EVEROLIMUS LEUCOVORIN CETUXIMAB GEMCITABINE TAMOXIFEN
h
